# Noninvasive Vagus Nerve Electrical Stimulation for Immune Modulation in Sepsis Therapy

**DOI:** 10.1021/jacs.4c16367

**Published:** 2025-03-04

**Authors:** Cam-Hoa Mac, Giang Le Thi Nguyen, Dien Thi My Nguyen, Sheng-Min Huang, Hsu-Hsia Peng, Yen Chang, Shih-Kai Lo, Hui-Hua Kenny Chiang, Yuan-Zhen Yang, Hsiang-Lin Song, Wei-Tso Chia, Yu-Jung Lin, Hsing-Wen Sung

**Affiliations:** † Department of Chemical Engineering, National Tsing Hua University, Hsinchu 300044, Taiwan; ‡ Department of Pharmacology, College of Medicine, National Cheng Kung University, Tainan 701401, Taiwan; § Department of Biomedical Engineering and Environmental Sciences, National Tsing Hua University, Hsinchu 300044, Taiwan; ∥ Taipei Tzu Chi Hospital, Buddhist Tzu Chi Medical Foundation and School of Medicine, Tzu Chi University, Hualien 970473, Taiwan; ⊥ Institute of Biomedical Engineering, National Yang-Ming Chiao Tung University, Taipei 112304, Taiwan; # Department of Pathology, National Taiwan University Hospital, Hsinchu Branch, Hsinchu 302058, Taiwan; ∇ Department of Orthopedics, National Taiwan University Hospital, Hsinchu Branch, Hsinchu 302058, Taiwan; ○ Research Center for Applied Sciences, Academia Sinica, Taipei 115201, Taiwan

## Abstract

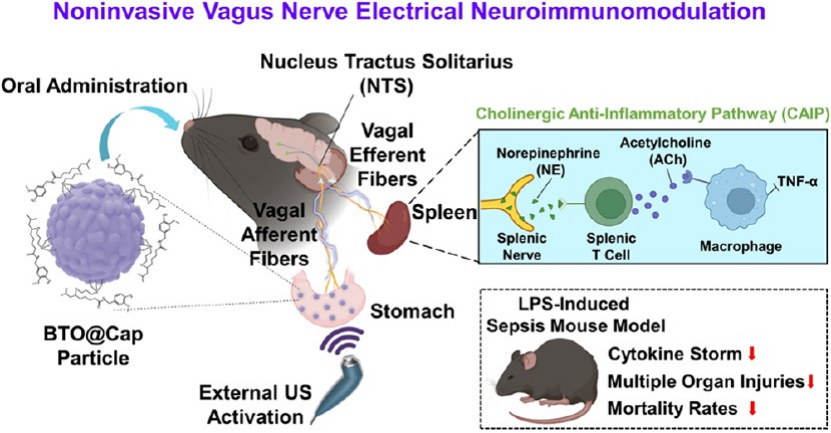

Sepsis presents a significant medical challenge due to its intense inflammatory response to infection, often resulting in high mortality rates. A promising therapeutic strategy targets the cholinergic anti-inflammatory pathway (CAIP), which regulates immune responses. This study investigates the ingestion of piezoelectric particles that adhere to the stomach lining, specifically targeting TRPV1 receptors. In a mouse model of sepsis, these particles, when activated by low-intensity pulsed ultrasound, generate mild electrical pulses. These pulses stimulate vagal afferent fibers, transmitting signals to the brain and modulating the neural-immune network via the CAIP. Consequently, this leads to a reduction in systemic inflammation, mitigating weight loss, alleviating multiple tissue injuries, and preventing death by modulating immune cells in the spleen. This approach addresses the critical need for noninvasive sepsis therapies, potentially improving patient outcomes. Utilizing portable ultrasound equipment with minimal thermal effects, this technique offers a safe and convenient treatment option, even for home use.

## Introduction

Sepsis, a serious medical condition marked by an extensive inflammatory response throughout the body, occurs when the body’s reaction to infection, especially bacterial infections, becomes out of control.^1−4^ Lipopolysaccharide (LPS), a primary constituent of bacterial cell walls, plays a significant role in driving the uncontrolled inflammation seen in the early stages of sepsis.^3,4^ During a serious bacterial infection, macrophages—key sentinel cells of the innate immune system—initiate a critical inflammatory cascade to eliminate the invading pathogen.^3,4^ LPS, located on the outer membrane of bacteria, directly engages Toll-like receptors on macrophages. This triggers a cytokine storm characterized by the rapid release of pro-inflammatory cytokines such as tumor necrosis factor-α (TNF-α), interferon-γ (IFN-γ), interleukin-1β (IL-1β), IL-6, and IL-17A into the bloodstream.^3−5^ This excessive inflammation not only harms cells and tissues but also significantly increases the risk of multiple organ injuries.

It is important to recognize, however, that while LPS-induced endotoxin shock mimics the early hyperinflammatory phase of sepsis,^6,7^ clinical sepsis is far more complex and dynamic.^8^ Most sepsis patients succumb not to the initial hyperinflammatory response but to secondary infections caused by compensatory immunosuppression and immunoparalysis, which are major contributors to sepsis-related deaths.^7,9^ Despite considerable efforts to improve treatment strategies, sepsis continues to be a significant medical challenge.

The cholinergic anti-inflammatory pathway (CAIP) plays a crucial role in the body’s innate defense against inflammation, finely coordinating responses to tissue damage or infection.^10−14^ This pathway involves communication between the nervous and immune systems, allowing peripheral nerves to regulate immune responses. Activation of the CAIP, whether through pharmacological means or electrical stimulation of the vagus nerve, has shown significant effectiveness in mitigating cytokine-mediated diseases.^10−14^ While pharmacological therapies offer targeted interventions, they may also cause unwanted side effects, which can limit their clinical application.^13^ Consequently, there has been an increasing emphasis on nonpharmacological approaches, particularly vagus nerve stimulation (VNS), which provides more precise and targeted stimulation.^5,13,14^

The conventional method of VNS involves implanting a stimulation electrode cuff on the left cervical vagus nerve, with the electrical generator positioned in the subcutaneous space of the left anterior chest.^15−18^ This invasive approach has been approved by the Food and Drug Administration (FDA) for the treatment of refractory epilepsy and depression. The neuroimmunomodulation effect of VNS is mediated through the CAIP, which regulates immune cells and dampens excessive inflammation, promoting the resolution of the inflammatory response. Electrical VNS is currently undergoing clinical trials for treating inflammatory diseases such as rheumatoid arthritis^15−17^ and colitis.^19,20^ However, the invasive nature of the stimulator implantation poses infection risks,^21,22^ along with reported side effects associated with direct VNS such as pain and temporary facial paresis,^12^ hindering its widespread adoption as a therapeutic approach.

Recently, a novel method utilizing noninvasive focused ultrasound (US) to stimulate the CAIP downstream of the vagus nerve in the spleen has been proposed to alleviate endotoxin-induced cytokine production.^12,23^ While promising, achieving precise targeting of nerve innervation within the organ using high-intensity focused US for safe and effective stimulation remains challenging.^12,23^ Therefore, there is an urgent need for a noninvasive and safe alternative to VNS for broad clinical application.

This study introduces a novel approach utilizing orally ingestible, US-activated piezoelectric particles designed to target and adhere to the gastric surface for noninvasive treatment of sepsis. The piezoelectric particles, composed of barium titanate (BaTiO_3_, BTO), are known for their ability to convert mechanical force into electricity. Previous research has explored BTO’s potential applications in bone replacement and repair.^24,25^ Furthermore, the BTO particles are chemically linked with capsaicin (Cap), a compound found in chili peppers, which serves as a ligand targeting the transient receptor potential vanilloid 1 (TRPV1) receptor, primarily situated on nerve endings of sensory neurons within vagal afferent fibers.^26−28^ TRPV1 receptors are also present in gastrin and parietal cells, as well as epithelial cells lining the stomach’s surface.^29−31^

**Figure 1:**
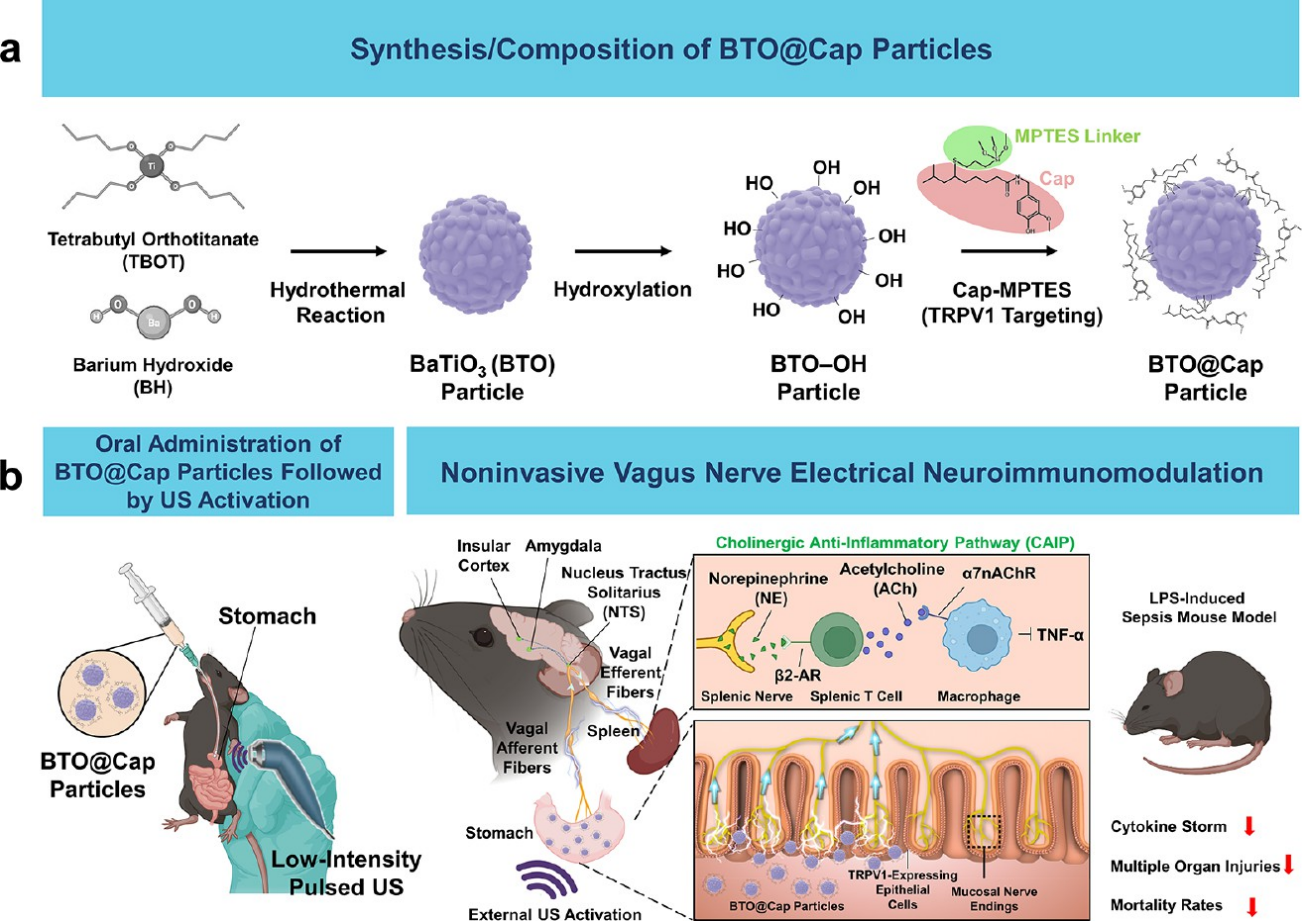
A noninvasive vagus nerve electrical stimulation system for immune modulation and its operating mechanism. (a) Synthesis and composition of BTO@Cap particles. (b) Upon oral administration, BTO@Cap particles adhere to the stomach lining, specifically targeting TRPV1 receptors. When exposed to low-intensity pulsed US, these particles generate mild electric pulses. These pulses stimulate vagal afferent fibers, which then transmit signals to the brain. This interaction modulates the neural-immune network via the CAIP, reducing systemic inflammation, multiple organ injuries, and mortality rates. As a result, this process significantly improves outcomes in mice with LPS-induced sepsis. Images were created with Biorender.com.

In a mouse model with LPS-induced sepsis, orally ingested piezoelectric BTO@Cap particles target and bind to Cap-sensitive TRPV1 receptors on the gastric surface (Figure 1). Upon exposure to low-intensity pulsed US irradiation using portable equipment, these particles (BTO@Cap/+US) generate mild electrical pulses. These pulses stimulate vagal afferent fibers traveling upward to the brainstem nuclei and relayed circuits, and downward to activate the efferent vagus nerve. This activation of the vagal efferent route triggers the CAIP, facilitating the release of acetylcholine (ACh). ACh binding to the alpha 7 nicotinic Ach receptor (α7 nAChR) on macrophages reduces their production of pro-inflammatory cytokines, thereby suppressing inflammation.^10−14,32,33^ This process effectively mitigates the inflammatory response and promotes the resolution of inflammation, tissue repair, and recovery. Through this mechanism, the CAIP helps to rebalance inflammation, contributing to overall immune homeostasis.

The proposed BTO@Cap/+US approach offers effective options in sepsis therapy with ease and efficiency, ultimately improving patient care outcomes. The low-intensity pulsed US utilized in the study ensures safe application by minimizing thermal impact. Its compact, portable design and user-friendly interface enhance reliability and accessibility, even in settings lacking trained medical personnel, such as at home.

## Results and Discussion

### Characteristics of BTO@Cap Particles

In this study, BTO particles were synthesized using a hydrothermal reaction, then surface-conjugated with Cap to form BTO@Cap particles. BTO is a well-known piezoelectric material with a high piezoelectric coefficient, biocompatibility, and stability across a wide range of temperatures and pH conditions, making it a promising candidate for various biomedical applications.^24,25,34^ Cap activates TRPV1 receptors,^26^ which are primarily found on sensory neurons but are also present in gastric epithelial cells.^27,29−31^

To prevent Cap from shedding or leaking from BTO particles in the harsh gastrointestinal (GI) environment, Cap was covalently attached to the particle surface, as detailed in our previous study.^35^ This process began by modifying Cap’s lipophilic tail with the silane coupling agent 3-mercaptopropyltriethoxysilane (MPTES) via a thiol–ene ‘click’ reaction, resulting in Cap-MPTES. The functionalized Cap was then chemically grafted onto BTO particles that had been hydroxylated with trialkoxysilanes (BTO–OH). Silanes form strong covalent oxane bonds with particle surfaces, effectively bridging inorganic and organic components.^36^

**Figure 2:**
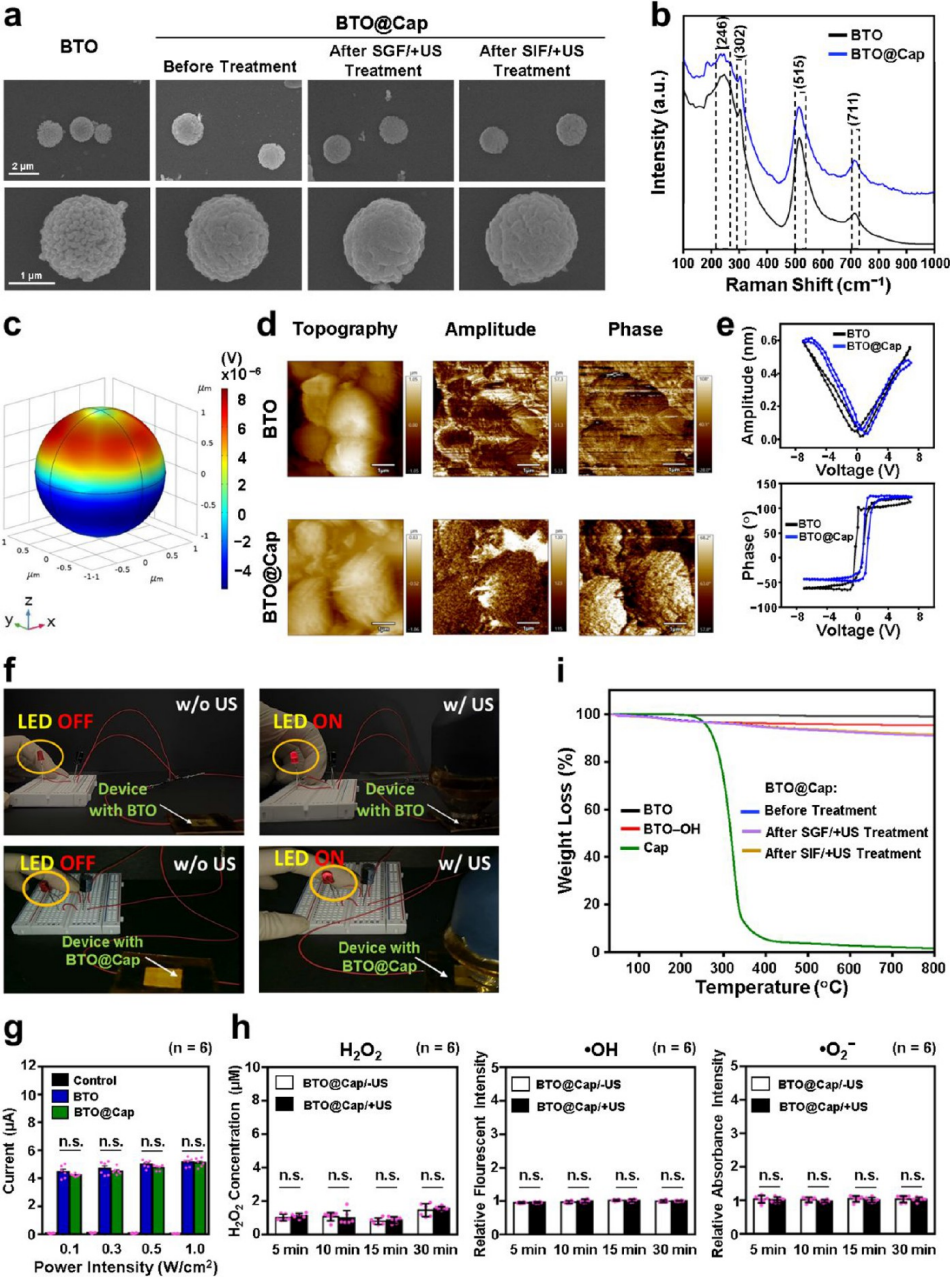
Characteristics of BTO@Cap particles. (a) SEM images of BTO particles and BTO@Cap particles before and after treatment in SGF or SIF under US stimulation. (b) Raman spectra of BTO and BTO@Cap particles. (c) COMSOL simulation of a single BTO particle under US irradiation at a power intensity of 0.3 W/cm^2^. (d) PFM images showing the topography, amplitude, and phase of BTO and BTO@Cap particles. (e) Piezoresponse amplitude–voltage and phase–voltage curves of BTO and BTO@Cap particles. (f) Photographs of the system with an LED bulb, demonstrating the illumination of the bulb when connected to an electromechanical device with BTO or BTO@Cap particles under US irradiation. (g) Measured current values of electrical outputs from the electromechanical device, comparing outputs without particles and with BTO or BTO@Cap particles at different power intensities. (h) Local levels of H_2_O_2_, ·OH, and ·O_2_^–^ detected in PBS containing BTO@Cap particles with or without US stimulation over various time periods. (i) TGA curves of BTO particles, BTO–OH particles, Cap, and BTO@Cap particles before and after treatment in SGF or SIF under US stimulation. Each dot represents one observed data point. n.s., not significant (*P* > 0.05).

The morphologies of the synthesized BTO and BTO@Cap particles were examined by scanning electron microscopy (SEM). As shown in Figure 2a, the BTO particles had a spherical shape with irregular protruding structures on their surfaces, with diameters ranging from 1.5 to 2.0 μm. After conjugation with Cap, the borders of the irregular protruding structures on the BTO particles became obscured. Nonetheless, the size of the resulting modified particles (BTO@Cap) remained consistent with that of the original BTO particles, measuring approximately 1.5–2.0 μm. In Figure S1a, the transmission electron microscopy (TEM) image of a single BTO particle shows consistent morphology with the SEM images. The selected area electron diffraction (SAED) patterns obtained from different positions within the same BTO particle are identical, confirming the single-crystalline nature of the particle.

The tetragonal and cubic phases are commonly observed phases of BTO, known for their distinct properties, particularly in piezoelectric behavior.^37,38^ Specifically, the tetragonal phase exhibits enhanced piezoelectric efficiency attributed to its asymmetrical crystal structure, which enables greater polarization and stronger piezoelectric properties. Raman spectroscopy has emerged as a preferred technique for analyzing crystallinity and phase composition in various materials.^39,40^ By employing this highly sensitive method, the cubic-tetragonal symmetry of synthesized BTO and BTO@Cap particles was determined. As illustrated in Figure 2b, the detected peaks at 246, 302, 515, and 711 cm^–1^ corresponded to the [A1(TO)], [B1, E(TO + LO)], [E(TO), A1(TO)], and [E(LO), A1(LO)] types of vibrational scattering, respectively. These peaks consistently match reported tetragonal BTO Raman scattering patterns,^39,40^ suggesting that the synthesized BTO particles exhibited favorable piezoelectric properties due to their tetragonal phases. Additionally, the high crystallinity of the BTO particles is evidenced by their sharp XRD peaks, which closely match the simulated perovskite structure (Figure S1b). Notably, the enlarged area exhibits doublet splitting at 2θ ≈ 45°, indicating that the BTO particles are crystallized in a tetragonal perovskite structure.^39,40^

### Piezoelectric Properties of BTO Particles

The COMSOL Multiphysics software was used to simulate the capability of the piezopotential generation of a BTO particle under US activation along the *z*-axis.^39,41,42^ The simulation results illustrate the distribution of electrical potential generated on the surface of the BTO particle (Figure 2c). This finding suggests that mechanical energy can be converted into electrical energy through US stimulation, implying potential applications of BTO particles in US-activated electrical generation. Specifically, under 0.3 W/cm^2^ US activation, it is estimated that a BTO particle with a diameter of 1.5 μm can generate a maximum surface piezopotential of 8 μV.

To delve deeper into the piezoelectric properties of the synthesized BTO and BTO@Cap particles, piezoresponse force microscopy (PFM) was employed. Figure 2d exhibits the topography, amplitude, and phase images of the tested samples. The AFM topography images depict the distinctive morphology of the BTO and BTO@Cap particles, each measuring approximately 1.5 μm. These findings align well with their corresponding amplitude and phase images.

The PFM amplitude and phase data allow for probing the local domain structure and polarization of the particles.^39,42,43^ The amplitude loop, reflecting the electric field-induced strain behavior, reveals their piezoelectric properties. Concurrently, the phase–voltage curves demonstrate the polarity switching property. As illustrated in Figure 2e, both BTO and BTO@Cap particles exhibited butterfly shaped loops under a voltage range of −7 to +7 V, indicating successive strain induced by the external electric field and confirming their robust local piezoelectric response. Furthermore, the phase–voltage signal of the particles showed a nearly 180° phase shift, signifying that changes in the applied external electric field can switch the local polarization of the particles. These findings suggest that the synthesized BTO and BTO@Cap particles possess a resilient local switching of piezoelectric polarization.

To further study their piezoelectric properties, the BTO and BTO@Cap particles were separately attached to an electromechanical device and exposed to US using a setup connected to a full-wave bridge-rectifier circuit, which was used to charge a capacitor and illuminate a light-emitting diode (LED) bulb (see Figure S2a). In Figure 2f, it is evident that in the absence of US irradiation, the LED bulb connected to the device with the test particles remained unlit. However, upon US irradiation, the LED bulb promptly illuminated, demonstrating the ability of these particles to generate an electrical current when exposed to US, as the US deformed these particles.

Subsequently, the currents generated by the BTO and BTO@Cap particles under various US power intensities were measured. As depicted in Figure 2g, an increase in US power intensity corresponded to a steady rise in the electrical current generated, underscoring the effective piezoelectric effect of the test particles. Notably, there were no significant differences in the electrical currents generated between the BTO and BTO@Cap particles under US activation at varying power intensities, suggesting that the conjugation of Cap on BTO particles did not impact their piezoelectric responses.

To further simulate in vivo conditions, an ex vivo current measurement experiment was conducted.^44,45^ In this setup (Figure S2b), the test particles were coated onto fluorine-doped tin oxide (FTO) glass, connected to a measurement device, and immersed in an electrolyte solution to mimic body fluids. US waves were applied from an approximately 1 cm distance through the glass wall of a beaker, simulating the stomach environment where US waves must penetrate fluid to activate the particles. The results (Figure S2c) showed that both BTO@Cap and BTO particles generated significant electrical currents compared to the US-alone control group, confirming that the treatment effectively generates electrical currents in a setting that approximates in vivo conditions.

### ROS Generation by Piezoelectric Effect of BTO@Cap Particles

Piezoelectric materials have a unique ability to generate electric charges when mechanically stressed, such as by exposure to US, which can initiate redox reactions with local water and dissolved oxygen, leading to the production of harmful reactive oxygen species (ROS) like hydrogen peroxide (H_2_O_2_), hydroxyl radical (·OH), and superoxide free radical (·O_2_^–^).^37,41^ To explore this effect, ROS levels in phosphate-buffered saline (PBS) were measured after applying US to BTO@Cap particles (4 mg/mL) at different power intensities. H_2_O_2_ was quantified using the Amplex Red Hydrogen Peroxide/Peroxidase Assay Kit,^46^ while ·OH and ·O_2_^–^ were detected using terephthalic acid (TA) and XTT, respectively.^46,47^ As shown in Figure S3, significant ROS production occurred when US intensity exceeded 0.3 W/cm^2^. Therefore, a power intensity of 0.3 W/cm^2^ was selected for VNS in sepsis treatment to avoid excessive ROS generation and ensure biosafety.

ROS generation by BTO@Cap particles following varying durations of US exposure at an intensity of 0.3 W/cm^2^ (BTO@Cap/+US) was also examined. The conditions used were derived from a subsequent in vitro safety study involving the use of US to activate BTO@Cap particles. Controls were conducted in the absence of US exposure (BTO@Cap/–US). The results depicted in Figure 2h reveal that the local levels of H_2_O_2_, ·OH, and ·O_2_^–^ generated in PBS in the BTO@Cap/+US groups were comparable to those in the corresponding BTO@Cap/–US control groups throughout the study. This suggests that the piezoelectric effect of the US-activated BTO@Cap particles is unlikely to produce significant harmful ROS.

### Stability of BTO@Cap Particles in GI Conditions

After oral administration, BTO@Cap particles face the harsh conditions of the GI tract, especially when exposed to US activation. To assess their stability under US activation within simulated GI conditions in vitro, the particles were individually incubated in simulated gastric fluid (SGF) and intestinal fluid (SIF) at 37 °C and exposed to US (0.3 W/cm^2^, three 5 min US doses with 5 min intervals between doses). Notably, the BTO@Cap/+US particles subjected to SGF or SIF treatments displayed similar sizes and morphologies to those not subjected to such treatments, as illustrated in Figure 2a.

To further evaluate stability, thermogravimetric analysis (TGA) was employed to measure the surface-grafted Cap contents before and after SGF or SIF treatments with US stimulation (0.3 W/cm^2^, three 5 min US doses with 5 min intervals between doses). The results presented in Figure 2i indicate that the Cap contents on the BTO@Cap/+US particles remained comparable before and after exposure to SGF or SIF, at approximately 4%. These findings strongly suggest that BTO@Cap particles can remain stable while passing through the GI tract, even when exposed to US.

### Safety of Using US in Activating BTO@Cap Particles

The safety of using US to activate BTO@Cap particles, which act as piezoelectric stimulators for VNS, in local tissue is crucial and depends heavily on the intensity and duration of exposure. In routine clinical practice, US is typically administered at power intensities ranging from 0.03 to 1.0 W/cm^2^.^48,49^ However, for more focused therapeutic applications, higher power intensities ranging from 10 to 10,000 W/cm^2^ may be used, potentially generating local heat.^49,50^ While this heating can be advantageous in certain therapeutic settings, such as thermal ablation of tumors, excessive heating can harm surrounding healthy tissue.^49,50^ Thus, ensuring the appropriate power intensity and duration of US exposure is vital for minimizing adverse effects and optimizing therapeutic outcomes.

**Figure 3:**
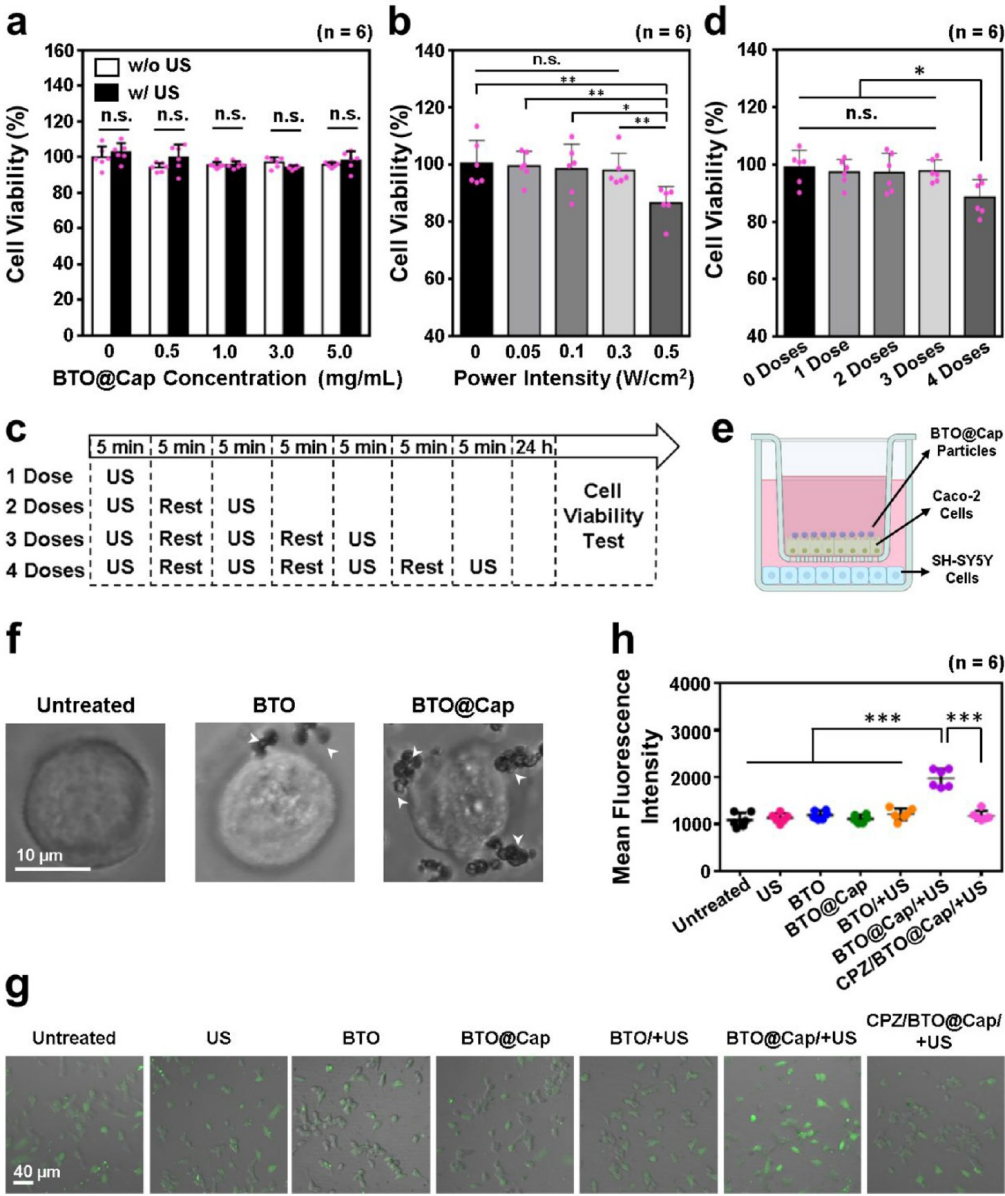
In vitro safety and potential of using BTO@Cap/+US for activating VNS. (a) Cytotoxicity assessment of BTO@Cap particles at various concentrations with and without US irradiation. (b) Assessment of cytotoxicity for BTO@Cap particles at a concentration of 4 mg/mL under varying power intensities of single-dose US irradiation. (c) Schematic representation of the US treatment schedule for different doses. (d) Evaluation of cytotoxicity for BTO@Cap particles at a concentration of 4 mg/mL under various doses of US irradiation at a power intensity of 0.3 W/cm^2^. (e) Schematic representation of the transwell model (created with Biorender.com). (f) Representative microscope images of Caco-2 cells illustrating the adsorption of BTO or BTO@Cap particles onto the cell membranes, indicated by white arrowheads, rather than internalization. (g) Fluorescence images depicting Ca^2+^ influx in SH-SY5Y cells following various treatments. (h) Quantification of mean fluorescence intensity representing intracellular Ca^2+^ signal through analysis of fluorescence images. Each dot represents one observed data point. *(*P* < 0.05), **(*P* < 0.01), and ***(*P* < 0.001); n.s., not significant (*P* > 0.05).

To assess the safety of using US to activate BTO@Cap particles, in vitro cell viability tests were conducted using Caco-2 cells, a commonly employed human epithelial cell line for assessing oral absorption in the GI tract.^51^ These cells were exposed to varying concentrations of BTO@Cap particles, both without US exposure (BTO@Cap/–US) and with exposure to pulsed US at an intensity of 0.3 W/cm^2^ (BTO@Cap/+US) for 5 min. The results indicate that neither the BTO@Cap/–US group nor the BTO@Cap/+US group caused significant toxicity toward Caco-2 cells at the different particle concentrations tested, compared to untreated cells (Figure 3a).

Furthermore, the impact of US power intensity on cell viability was investigated using BTO@Cap particles at a concentration of 4 mg/mL for 5 min. Figure 3b illustrates that the viability of cells treated with BTO@Cap/+US at power intensities up to 0.3 W/cm^2^ remained unaffected. Moreover, the cell viability following multiple doses of BTO@Cap/+US treatment at 0.3 W/cm^2^ was examined, with a 5 min break between each dose (Figure 3c). As depicted in Figure 3d, the viability of cells treated with three doses of BTO@Cap/+US remained comparable to that of untreated cells and those receiving one or two doses of BTO@Cap/+US. In contrast, those treated with four doses of BTO@Cap/+US showed some cytotoxicity. Based on these findings, a three-dose US treatment schedule at a power intensity of 0.3 W/cm^2^ was adopted to evaluate the efficacy of BTO@Cap/+US in generating piezoelectricity for VNS in subsequent studies.

### Potential of BTO@Cap/+US for Initiating VNS

Gastric epithelial cells line the inside of the stomach, while vagal afferent endings are spread throughout its various layers, often reaching into the lamina propria where they can interact with these epithelial cells.^52,53^ To send electrical stimulation signals from the stomach to the brain, BTO@Cap particles need to attach to the gastric epithelial cells first. When activated by US irradiation, these particles produce piezoelectricity, which is then transmitted to the vagal afferent endings below. This process takes place within the gastric fluid, which contains electrolytes, playing a crucial role in initiating VNS.

To examine whether BTO@Cap particles remain on the cell membranes or undergo internalization, the particles were labeled with Alexa Fluor 633 (f-BTO@Cap) and cultured with Caco-2 cells. Untreated cells and cells treated with f-BTO particles served as controls. After incubation, the cells were thoroughly washed with DPBS, stained with Hoechst (nuclear marker) and DiI (cell membrane marker), and observed using CLSM. The results reveal that no significant cellular uptake of BTO@Cap particles was detected. Instead, the particles appeared to adhere to the cell membrane (Figure S4).

The effectiveness of BTO@Cap/+US as piezoelectric stimulators for initiating VNS was investigated in vitro using a transwell culture model (Figure 3e). In this experiment, Caco-2 cells, known for their expression of high levels of TRPV1 receptors,^29,54^ were seeded in the upper chamber. Concurrently, SH-SY5Y-derived neuron-like cells, which exhibit heightened expression of voltage-gated calcium channels (VGCCs),^34,55^ were seeded in the lower chamber. SH-SY5Y-derived neuron-like cells are a cloned subline of a neuroblastoma cell line frequently utilized as a cellular model in laboratory settings to study specific aspects of neuronal biology and function.^34,55^ In the study, the following groups were used as controls: untreated, treated with BTO particles alone (BTO), BTO@Cap particles alone (BTO@Cap), US alone (US), BTO with US (BTO/+US), and BTO@Cap/+US with capsazepine (CPZ), a selective TRPV1 antagonist, pretreatment (CPZ/BTO@Cap/+US).

After incubating BTO@Cap particles with the upper layer of Caco-2 cells, adhesion occurred through the interaction between Cap on BTO particles and TRPV1 expressed on the Caco-2 cell membrane (Figure 3f). This finding aligns with observations from the CLSM image (Figure S4). Subsequently, the piezoelectric effect of BTO@Cap on SH-SY5Y-derived neuron-like cells was examined following US irradiation. VGCCs on SH-SY5Y neuron-like cells play crucial roles in regulating calcium ion (Ca^2+^) influx into cells in response to changes in membrane potential, leading to elevated intracellular Ca^2+^ concentrations. To detect this Ca^2+^ influx, the Ca^2+^-sensitive fluorescence probe Fluo-8 was utilized. Intriguingly, the intracellular Ca^2+^ concentration significantly increased only when SH-SY5Y neuron-like cells were treated with BTO@Cap/+US compared to the control groups (Figure 3g,h). This observation indicates that the piezoelectric effect of BTO@Cap/+US on Caco-2 cells in the upper chamber might activate SH-SY5Y neuron-like cells in the lower chamber within the culture medium containing electrolytes, suggesting its potential to initiate VNS.

### In Vivo Biodistribution

Following promising in vitro findings, the subsequent phase of the study centered on the assessment of in vivo biodistribution of orally administered BTO@Cap particles, along with their therapeutic efficacy as VNS stimulators when activated by low-intensity pulsed US. Initially, investigation was conducted into the ability of BTO@Cap particles to target and adhere to the gastric surface subsequent to oral ingestion in mice. For comparison purposes, counterparts lacking surface-conjugation of Cap (BTO particles) were utilized as controls. To minimize any interference from diet-related factors during imaging, the test mice were exclusively fed a low-fluorescent diet for 3 days before the experiment.

The experiment involved administering indocyanine green (ICG)-labeled test particles to mice via oral gavage after an overnight fast. ICG is a commonly used dye for near-infrared (NIR) fluorescence imaging.^56^ To address the possibility of fluorescent dye detachment under the strongly acidic conditions of gastric juice, an in vitro stability study was first conducted to evaluate the retention of the NIR signal in ICG-labeled-BTO particles. In this experiment, the particles were immersed in SGF, thoroughly mixed, and incubated for 2 h. After incubation, the particles were centrifuged to separate the solid phase from the SGF solution, and the NIR signal was analyzed using an imaging system. As a control, the NIR signal was also captured immediately after adding ICG-labeled-BTO particles to SGF and allowing them to settle. The results, presented in Figure S5, show no significant difference in the NIR signal intensity between the two groups. Additionally, no detectable NIR signal was observed in the supernatant solutions. These findings confirm that the fluorescent dyes remain securely bound to the particles, even under the acidic conditions of gastric fluid.

Subsequently, the stomachs of the mice were collected at different intervals after gavage, and NIR-II imaging was used to visualize the distribution of the particles on the gastric surfaces ex vivo. NIR-II imaging was chosen because it effectively reduces tissue autofluorescence, which is often encountered in the visible light region.^57^ This methodology enabled the assessment of particle localization and persistence in vivo, providing valuable insights into their potential as effective VNS stimulators.

**Figure 4:**
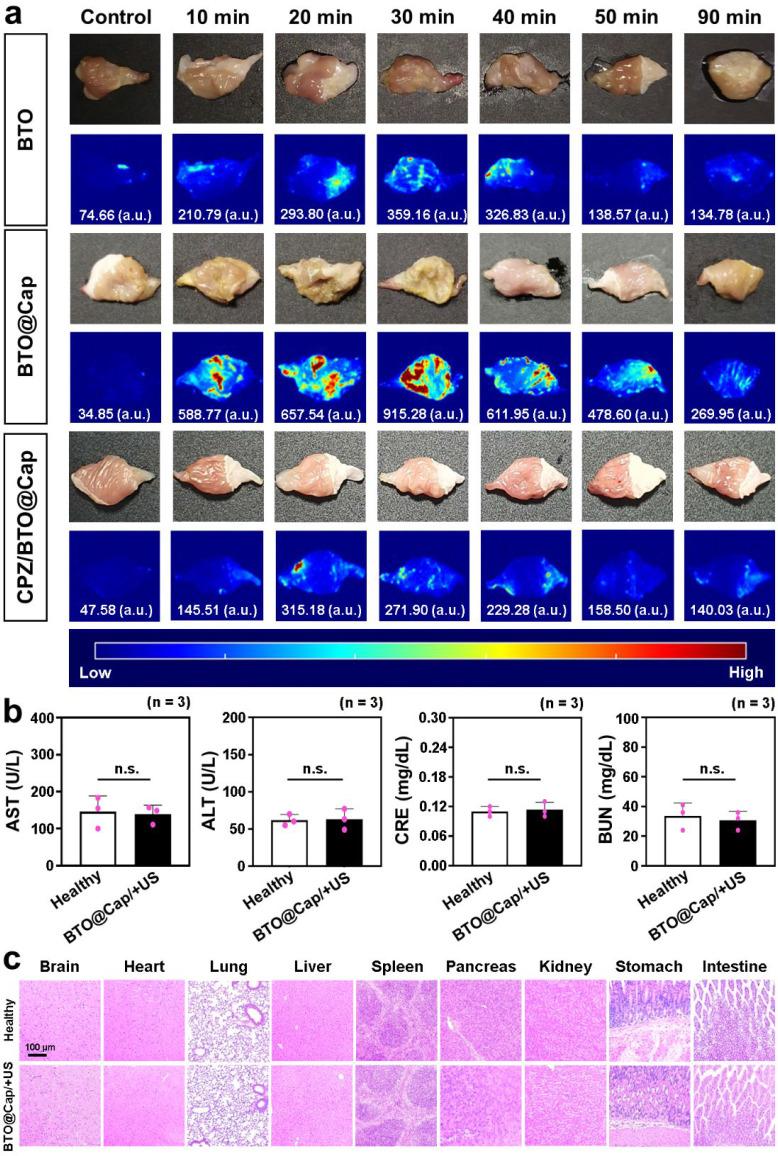
In vivo biodistribution and safety profile of orally ingested BTO@Cap particles. (a) Ex vivo NIR-II images depicting the biodistribution of ICG-labeled BTO and BTO@Cap particles on gastric surfaces at specific time intervals following oral administration, with CPZ pretreatment used as a TRPV1 inhibitor. The average fluorescence intensity on the gastric surface is quantified and presented below each image. (b) Serum levels of AST, ALT, CRE, and BUN in healthy mice and in mice treated with BTO@Cap/+US. (c) H&E staining images of major organs harvested from healthy mice and from mice treated with BTO@Cap/+US. Each dot represents a single data point. n.s., not significant (*P* > 0.05).

Following oral gavage, fluorescence signals emitted by the ICG-labeled particles were monitored on the gastric surface (Figure 4a). Over time, the area of the gastric surface displaying fluorescence signals increased, peaking at approximately 30 min postgavage for both groups under study. However, the area covered by fluorescence and its intensity were broader and more intense for the BTO@Cap-treated group compared to the BTO-treated group. These observations likely stem from the interaction between the ligand (Cap) on particles and the receptor (TRPV1) on gastric epithelial cells, leading to increased attachment of BTO@Cap particles to the gastric surface. Subsequently, the fluorescence area and intensity gradually diminished, indicating the detachment of some particles from the gastric mucosal surface, potentially due to mucus clearance. Regular mucus clearance aids in removing accumulated foreign substances.^58^

To further validate the targeting effect on TRPV1 receptors, CPZ was administered intraperitoneally prior to the oral administration of ICG-labeled BTO@Cap particles. As shown in Figure 4a, NIR-II imaging results from the CPZ/BTO@Cap group indicated that fluorescence intensity on the gastric surface was significantly reduced when TRPV1 channels were blocked by CPZ. This confirms that TRPV1-mediated targeting is critical for the adsorption of BTO@Cap particles.

To investigate whether orally ingested particles could enter the body, major visceral organs were retrieved and imaged ex vivo using an NIR-II camera at specific intervals after ingesting the ICG-labeled BTO@Cap particles. Figure S6a shows no significant fluorescence signals in the major visceral organs throughout the 24-h study period, indicating that orally ingested BTO@Cap particles did not breach the GI barrier and accumulate in the body. Instead, they were gradually expelled through fecal excretion, with complete elimination within 24 h (Figure S6b). These findings suggest that the microscale size of the BTO@Cap particles used in this study prevents their absorption during passage through the GI tract. Previous research has demonstrated that intestinal enterocytes can only absorb nanoscale particles ranging from 50 to 500 nm.^59,60^

### Thermal Effect and Biosafety

The thermal effect of BTO@Cap particles upon US activation was examined by irradiating the stomachs harvested 30 min after oral ingestion. Pulsed US from portable equipment was applied at power intensities of up to 0.5 W/cm^2^ for 5 min in three doses, with a 5 min break between each dose. The surface temperature of the stomach was then recorded using an infrared thermal camera. As illustrated in Figure S7, only a slight increase in the local temperature on the gastric surface was observed (∼1.4 °C), indicating that the thermal impact of piezoelectric BTO@Cap particles activated under low-intensity US was insignificant.

To assess the biosafety of orally ingested BTO@Cap particles in mice following a three-dose US treatment schedule at a power intensity of 0.3 W/cm^2^ targeted at the stomach (denoted as BTO@Cap/+US thereafter), levels of serum aspartate transaminase (AST), alanine transaminase (ALT), creatinine (CRE), and blood urea nitrogen (BUN) were measured. Additionally, histological sections of their major visceral organs were examined. The healthy mice served as a control. Compared to the healthy mice, no significant changes in AST and ALT levels (markers of hepatic function) or CRE and BUN levels (markers for renal function) were observed following oral treatment with BTO@Cap/+US (Figure 4b). Furthermore, the results, depicted in Figure 4c, showed no inflammatory reactions in the experimental tissues compared to those of healthy mice. These findings collectively suggest the favorable safety of BTO@Cap/+US as VNS stimulators within the GI tract of mice.

An open field test was conducted to evaluate the locomotor activity of the experimental animals. As shown in Figure S8, the behavior of BTO@Cap/+US-treated mice was comparable to that of healthy mice, with no significant difference in the total distance traveled over 1 h between the two groups. These findings indicate that BTO@Cap/+US treatment did not impair overall locomotor activity and had no adverse effects on brain function. Additionally, TRPV1 receptors are involved in various physiological processes, and their activation could potentially lead to unintended off-target effects or discomfort. However, Figure S8 shows that the mice exhibited normal exploratory behaviors post-treatment, including active navigation of both corners and peripheral areas of the test environment. These observations suggest no evidence of anxiety, altered pain perception, or thermoregulatory disturbances, indicating that the treatment is well-tolerated.

### Therapeutic Efficacy

The therapeutic efficacy of combining oral BTO@Cap particles with US in a three-dose treatment regimen (BTO@Cap/+US) was assessed in a sepsis mouse model. To induce the sepsis model, mice were administered intraperitoneal injections of either a lethal dose (20 mg/kg) or a nonlethal dose (10 mg/kg) of LPS. Endotoxin release, particularly LPS from bacterial infections, is responsible for the majority of clinical sepsis cases.^6,61^ In research, LPS is frequently administered to animals to induce systemic inflammation that mimics the pathophysiology of sepsis, making it a widely used model for investigating sepsis mechanisms and evaluating potential therapeutic interventions.^2−4^ Subsequently, the mice were divided into different treatment groups, with healthy mice serving as the control group for comparison.

**Figure 5:**
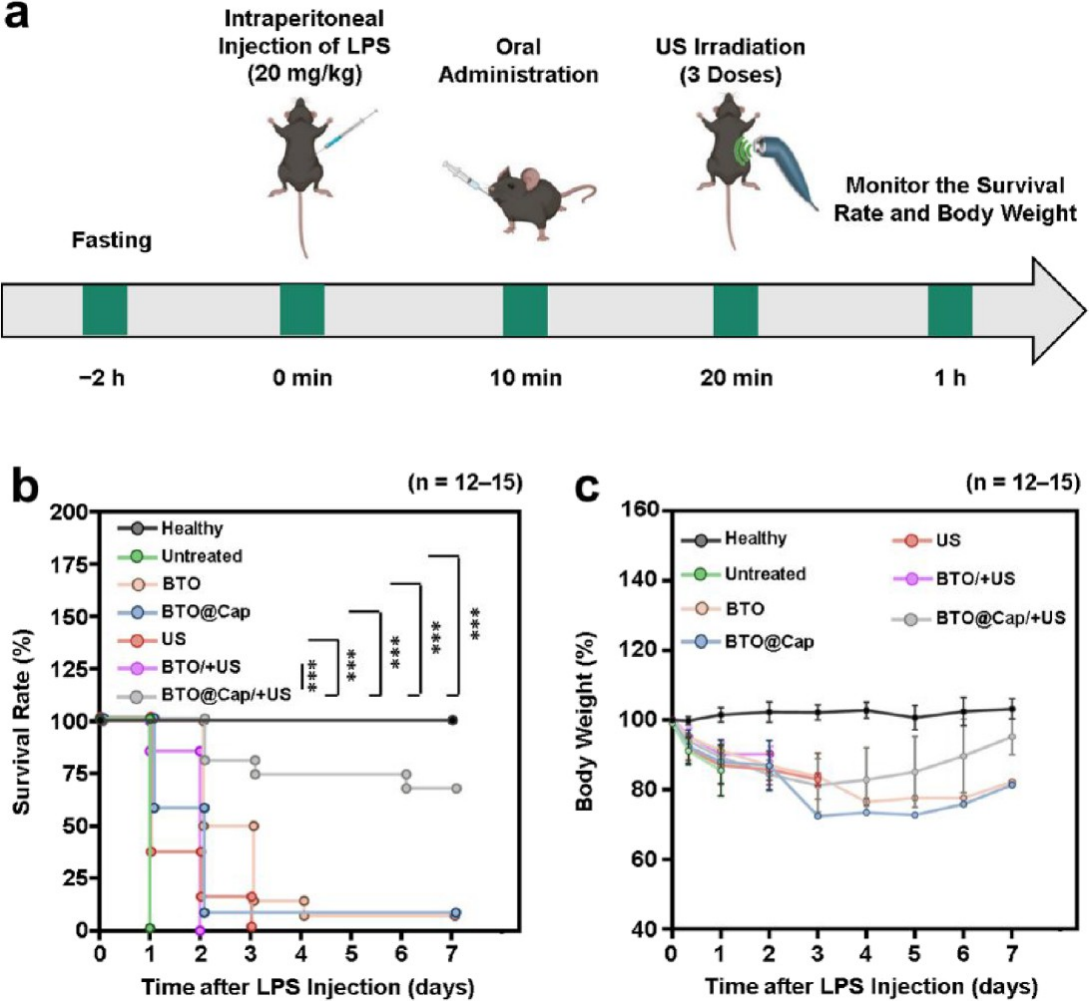
Therapeutic efficacy on survival rate and body weight. (a) Schematic timeline and treatment protocol for studying survival rate and body weight in LPS-induced mice (created with Biorender.com). (b) Survival rate and (c) body weight of healthy mice and LPS-induced septic mice after different treatments. ***(*P* < 0.001).

At the lethal dose of LPS, the survival rate and body weight of the mice were monitored for seven consecutive days (Figure 5a). In sepsis, LPS binds to Toll-like receptors on immune cells, particularly macrophages, triggering a massive release of inflammatory cytokines, known as a cytokine storm.^1−4^ Failure to promptly clear these cytokines leads to irreversible damage to cells and tissues, culminating in multiple organ failure and death. Hence, it is crucial to devise therapeutic approaches that can effectively reduce systemic inflammation and enhance survival rates in patients with sepsis.

The results showed that all untreated mice died within the first 24 h. By day seven, mice treated with BTO@Cap/+US exhibited a significantly higher survival rate of 67% (Figure 5b), while the control groups (BTO, BTO@Cap, US, and BTO/+US) showed minimal survival. This improved survival rate in the BTO@Cap/+US-treated group is likely attributed to the combined effects of Cap’s targeting capability and the mild electrical pulses generated by the BTO particles, which were further enhanced by external US stimulation. Additionally, the surviving mice in the BTO@Cap/+US group experienced less weight loss and recovered more quickly compared to the control mice (Figure 5c). These findings suggest that BTO@Cap/+US not only improves survival rates in sepsis-affected mice but also reduces weight loss, facilitating faster recovery.

To assess the long-term effects of BTO@Cap/+US treatment, we monitored both survival rate and body weight of test mice for 21 days post-treatment. As shown in Figure S9a,b, the treated mice continued to survive beyond day 7 (compared to the data in Figure 5b), even after the treatment was discontinued. Notably, by day 8, the body weight of sepsis-affected mice treated with BTO@Cap/+US had significantly recovered, reaching levels comparable to those of healthy controls. These findings suggest that the treatment not only improves survival rates but also promotes recovery to a healthy state, even after cessation of the treatment.

In this study, mice received a single treatment with BTO@Cap and US following LPS administration. No additional LPS challenges were introduced, allowing the mice’s immune systems to recover and regain homeostasis. The observed long-term effects suggest that the therapy promotes immune recovery in the early stages of sepsis rather than inducing prolonged immune suppression.

**Figure 6:**
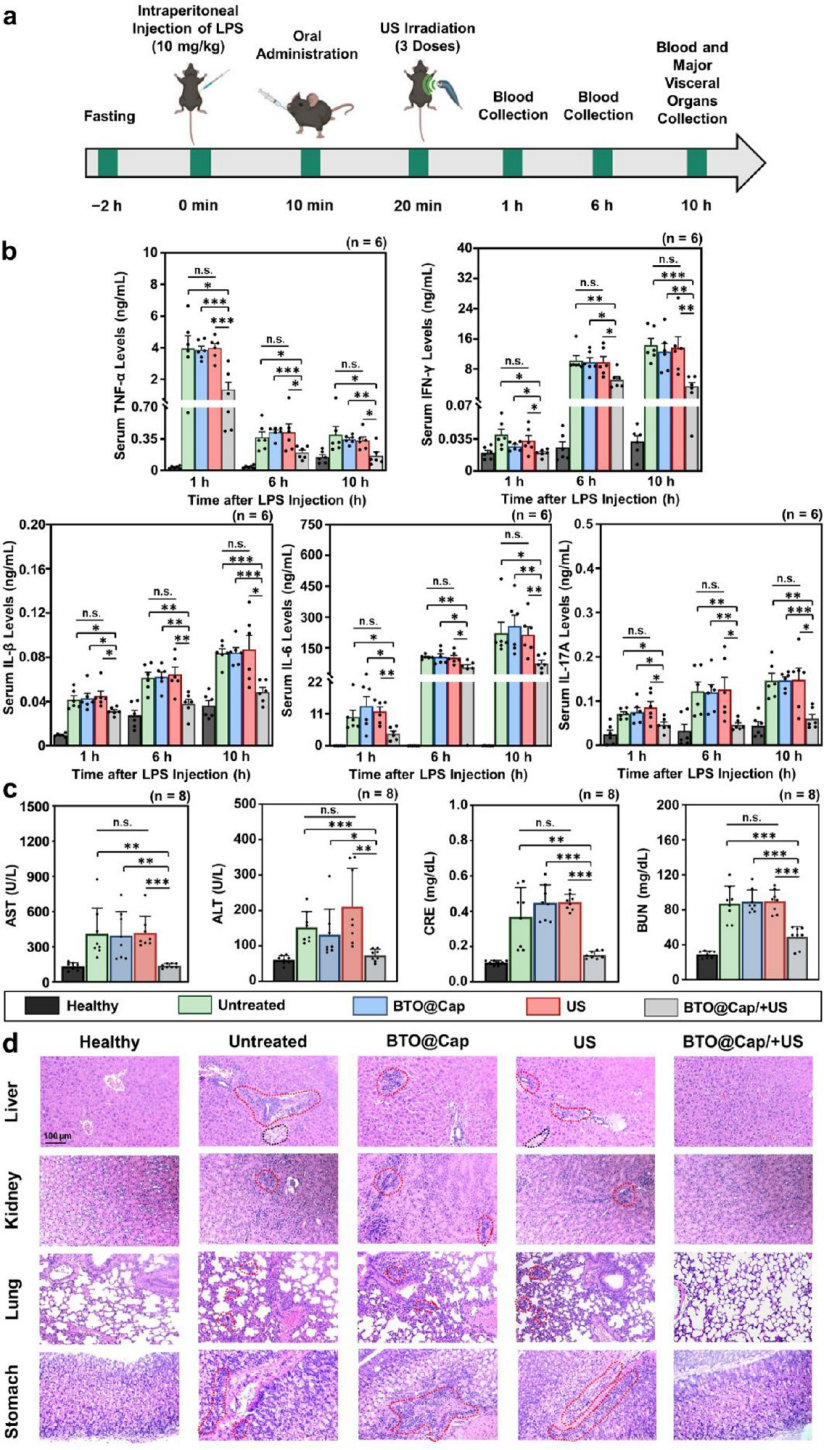
Therapeutic efficacy in mitigating cytokine storm and multiple organ injuries. (a) Schematic timeline and treatment protocol for studying pro-inflammatory cytokines and multiple organ injuries in LPS-induced septic mice (created with Biorender.com). (b) Serum levels of pro-inflammatory cytokines (TNF-α, IFN-γ, IL-1β, IL-6, and IL-17A) and (c) serum levels of AST, ALT, CRE, and BUN collected from healthy mice and septic mice after different treatments. (d) H&E staining images of liver, kidney, lung, and stomach tissues collected from healthy mice and septic mice after various treatments. Inflamed regions are outlined with red dashed lines, and necrotic regions are outlined with black dashed lines. Each dot represents one data point. *(*P* < 0.05), **(*P* < 0.01), and ***(*P* < 0.001); n.s.: not significant (*P* > 0.05).

At the nonlethal dose of LPS, attention was directed toward the changes in serum pro-inflammatory cytokines in mice at 1, 6, and 10 h post-treatment (Figure 6a). These changes are indicative of important characteristic features of sepsis that can reflect the severity of systemic inflammation and the progress of each treatment modality. At the end of the observation period, serum levels of AST, ALT, CRE, and BUN in mice were measured. Subsequently, the animals were sacrificed, and their major visceral organs were harvested and processed for histological examination.

Figure 6b illustrates that upon intraperitoneal introduction of LPS in mice (untreated), their levels of pro-inflammatory cytokines, including TNF-α, IFN-γ, IL-1β, IL-6, and IL-17A, significantly increased compared to healthy mice, as anticipated. Neither treatment with BTO@Cap alone nor US alone showed significant effects in reducing these pro-inflammatory cytokines at the investigated intervals. However, the combination of BTO@Cap/+US demonstrated significant effectiveness in reducing the production of pro-inflammatory cytokines. The levels of TNF-α and IL-1β showed no significant changes in mice treated with BTO, BTO/+US, or Cap alone (Figure S10). To further validate the findings, the nonpiezoelectric cubic BTO particles were synthesized using a similar procedure as tetragonal BTO particles, with modifications to the reaction temperature and duration. The synthesized nonpiezoelectric cubic BTO displayed an XRD pattern closely matching the simulated cubic crystalline structure of BTO (Figure S11a). Additionally, these particles exhibited minimal electrical current generation compared to the ultrasound-alone control group (Figure S11b). The cytokine results indicated that BTO@Cap particles synthesized using nonpiezoelectric cubic BTO (cubic BTO@Cap) could not suppress the cytokine increase in septic mice compared to the untreated group (Figure S10). Similar trends were observed in the measurement of serum levels of AST, ALT, CRE, and BUN in mice with LPS-induced sepsis at the end of the experiment. Only the combination treatment of BTO@Cap/+US effectively reduced serum levels of AST, ALT, CRE, and BUN (Figure 6c) and alleviated inflammation in major visceral organs (Figure 6d). This indicates that the treatment has the potential to mitigate multiple tissue injuries. Together, these findings suggest a potent anti-inflammatory effect of the BTO@Cap/+US approach in treating sepsis.

### Mechanism of Anti-Inflammatory Effect of BTO@Cap/+US

The anti-inflammatory effect of BTO@Cap particles may stem from their ability to target gastric epithelial cells through the interaction between the ligand (Cap) and receptor (TRPV1) (Figures 3f and 4a), as well as their capacity to generate piezoelectricity when activated by US (Figures 2c,f,g, and S2c). These mild electrical pulses may then activate neuron cells (Figure 3g,h), stimulating vagal afferent fibers. Subsequently, these fibers project to the nucleus tractus solitarii (NTS) in the brainstem, initiating the vagal efferent fibers via the CAIP, and ultimately relaying to the splenic nerve (Figure 1). This pathway relies on a robust neural-immune interaction to regulate immune function and mitigate the inflammatory response to infection.^11−14,23^

**Figure 7:**
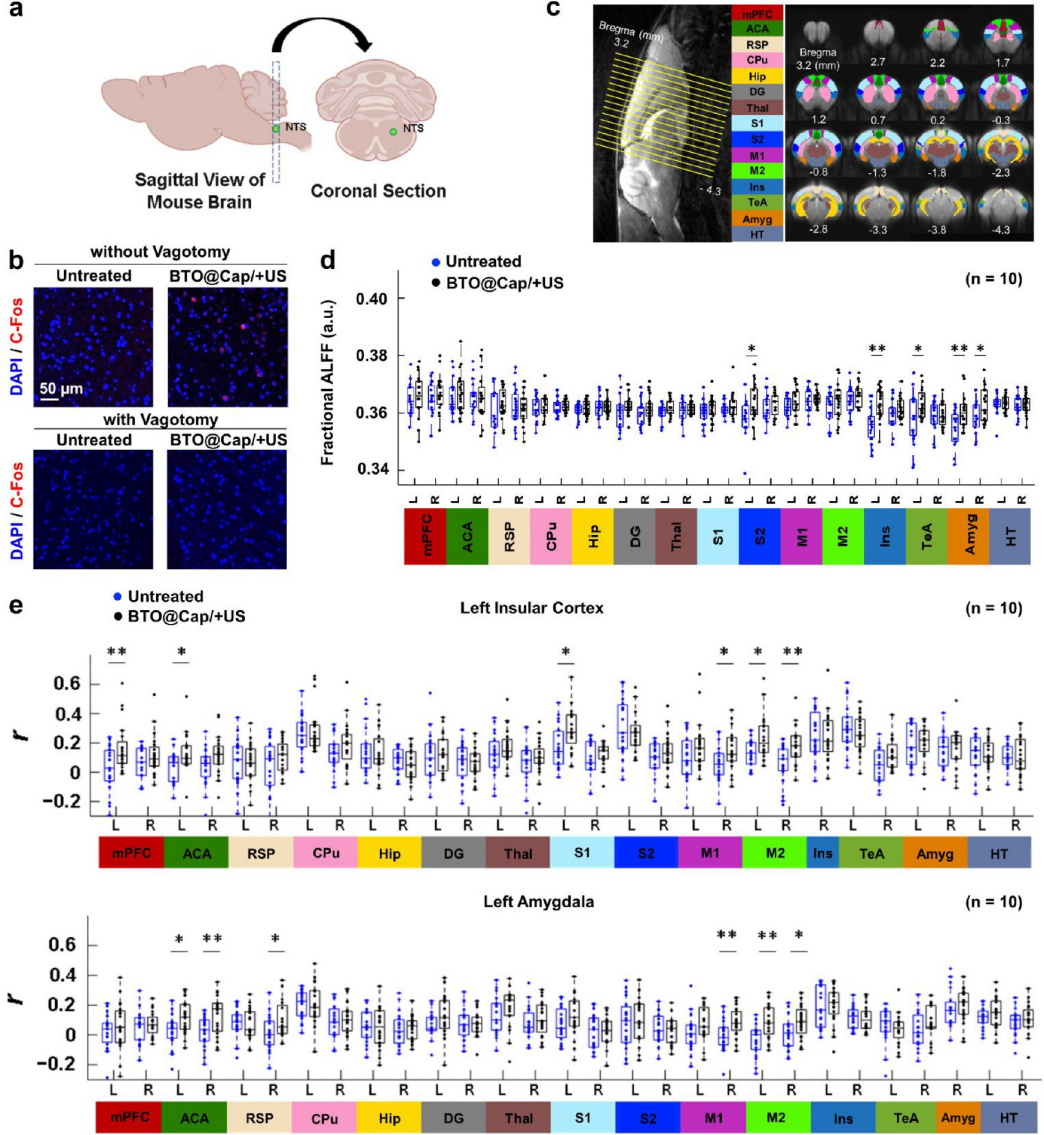
Brain-specific mechanism underlying the anti-inflammatory effects. (a) Schematic diagrams illustrating the location of the NTS in the brainstem (created with Biorender.com). (b) Immunofluorescence staining images depicting c-Fos expression in the area defined as the NTS in septic mice, without or with vagotomy, before and after treatment with BTO@Cap/+US. (c) Illustration of the selected brain regions for analyzing rs-fMRI data. (d) Results of fALFF analysis in the selected brain regions of the two mouse groups. (e) Functional connectivity between the left insular and left amygdala with other selected brain regions, indicated by Pearson’s correlation coefficient (*r*). mPFC, medial prefrontal cortex; ACA, anterior cingulate area; RSP, retrosplenial area; CPu, caudate putamen; Hip, hippocampus; DG, dentate gyrus; Thal, thalamus; S1, primary somatosensory cortex; S2, secondary somatosensory cortex; M1, primary motor cortex; M2, secondary motor cortex; Ins, insular cortex; TeA, temporal association area; Amyg, amygdala; HT, hypothalamus. Each dot represents one observed data point. *(*P* < 0.05), and **(*P* < 0.01).

To directly assess the activation of NTS cells by BTO@Cap/+US, the level of c-Fos, a neuronal activity marker,^23,34^ in the NTS of mouse brains was visualized through immunolabeling (Figure 7a). This analysis was conducted in both untreated sepsis mice and those treated with BTO@Cap/+US. In Figure 7b, it is evident that mice treated with BTO@Cap/+US showed a notable increase in c-Fos^+^ cells in the NTS area of the brainstem compared to untreated mice.

Additionally, vagotomy was performed prior to sepsis induction and treatment administration. In these mice, BTO@Cap/+US stimulation failed to activate the NTS, as shown by the absence of c-Fos^+^ cells in the NTS (Figure 7b). This finding underscores that the activation of the NTS in the brainstem by BTO@Cap/+US is mediated through the vagus nerve. The NTS is essential for relaying vagal afferent signals to higher brain regions, including the forebrain.^14,23^

The investigation of the regions where the forebrain was activated by the BTO@Cap/+US generated electrical pulses in mice was conducted using resting-state functional magnetic resonance imaging (rs-fMRI). This technique is widely used to monitor brain connectivity and networks, as well as the changes they undergo following VNS.^62−64^ The control group consisted of untreated sepsis mice. Measures commonly employed in rs-fMRI, such as fractional amplitude of low-frequency fluctuation (fALFF) and degree centrality, were utilized to investigate the functional differences in the forebrain between untreated mice and those treated with BTO@Cap/+US. Figure 7c presents 16 consecutive slices of brain functional images in the transversal view, accompanied by their corresponding regions of interest (ROIs) used for analyzing rs-fMRI signals. The complete names of each ROI listed in Figure 7c are provided in the caption.

Compared to the untreated mice, elevated fALFF was observed in the left S2, left insular cortex, left TeA, and bilateral amygdala in the BTO@Cap/+US-treated mice (Figure 7d). Figure S12a illustrates the averaged correlation matrices of the two groups of mice, while Figure S12b displays the degree centrality calculated from the correlation matrices of each brain region in both groups. In comparison with the untreated mice, the BTO@Cap/+US-treated mice presented increased degree centrality in the left insular cortex and left amygdala. Since the results of both fALFF and degree centrality measures concurrently indicated significant differences between the two groups in the left insular cortex and left amygdala, the functional connectivity of these regions with other selected brain regions was exclusively presented. Figure 7e reveals that the BTO@Cap/+US-treated mice exhibited a significantly higher correlation coefficient (*r*) than the untreated mice between the left insular cortex and left mPFC, left ACA, left S1, right M1, and bilateral M2. Meanwhile, the BTO@Cap/+US-treated group also demonstrated a higher *r* than the untreated mice between the left amygdala and bilateral ACA, right RSP, right M1, and bilateral M2, as depicted in Figure 7e. These results illustrate the response of the forebrain to vagal stimulation with BTO@Cap/+US treatment.

When the NTS in the brainstem is stimulated, it activates regions in the forebrain, such as the amygdala and insular cortex, along with the CAIP. Although this process begins with parasympathetic signaling through the efferent vagus nerve, it ultimately engages catecholaminergic fibers from the splenic sympathetic nerve (Figure 1).^10−14,23,65,66^ This interaction triggers the release of norepinephrine (NE), a key neurotransmitter in sympathetic signaling, within the spleen. The released NE binds to beta-2 adrenergic receptors (β2-AR) on a specific subset of splenic T cells that express choline acetyltransferase (ChAT) to produce Ach.^10−14^ The ACh then binds to a7 nAChR on macrophages. This interaction results in a significant reduction in the production of pro-inflammatory cytokines, particularly TNF-α, by these cells. TNF-α is considered the primary cytokine mediating the inflammatory response observed in sepsis.^67^

**Figure 8:**
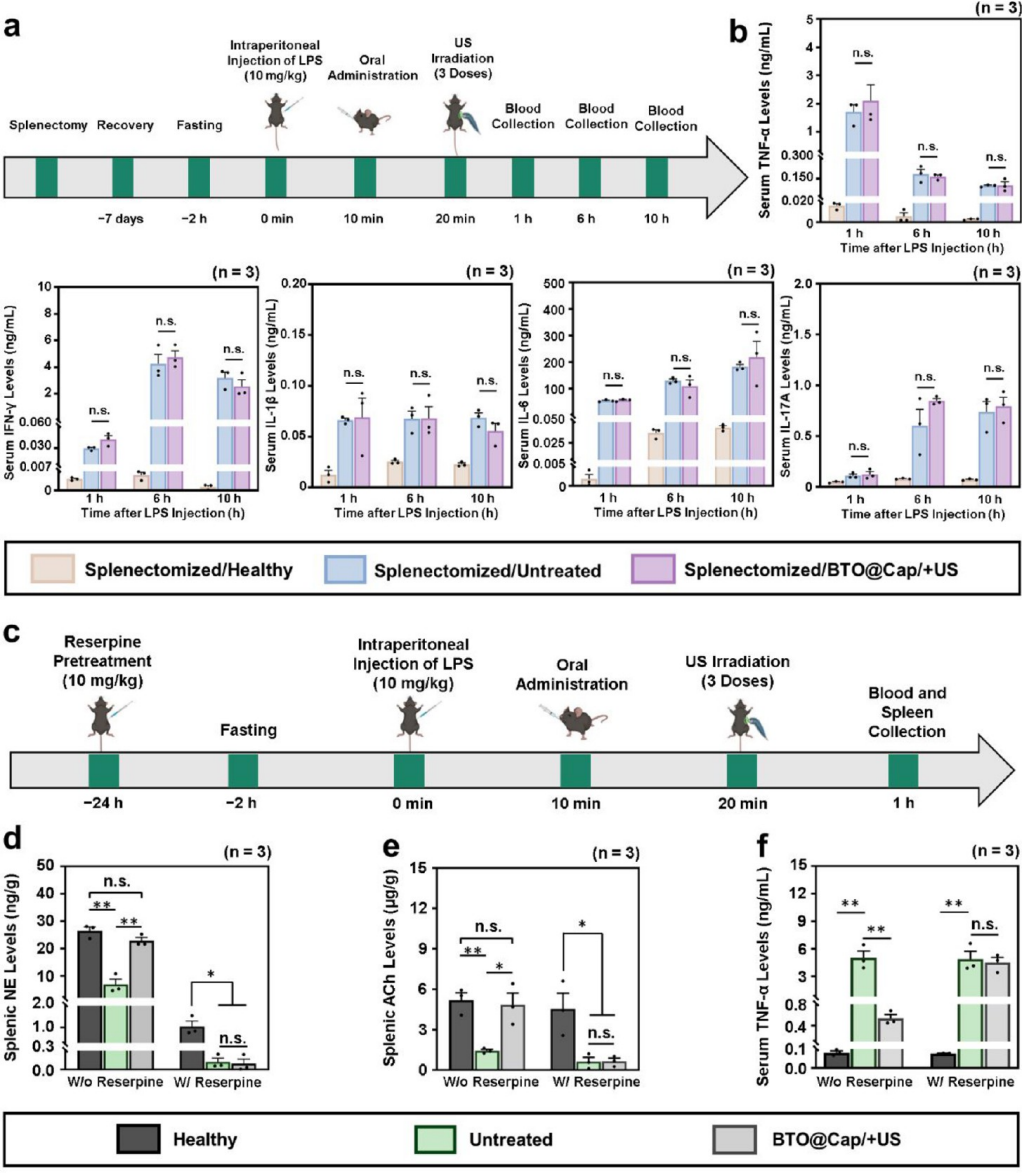
Spleen-specific mechanism underlying the anti-inflammatory effect. (a) Schematic timeline illustrating the treatment protocol for studying the role of spleen in CAIP in the LPS-induced septic mice (created with Biorender.com). (b) Serum levels of pro-inflammatory cytokines (TNF-α, IFN-γ, IL-1β, IL-6, and IL-17A) collected from healthy mice and septic mice that underwent splenectomy after various treatments. (c) Schematic timeline illustrating the treatment protocol for studying the CAIP in the spleen in the LPS-induced septic mice (created with Biorender.com). (d) Splenic NE, (e) Ach, and (f) serum TNF-α levels in healthy mice and septic mice before and after BTO@Cap/+US treatment. Reserpine pretreatment was used to deplete catecholamines. *(*P* < 0.05), and **(*P* < 0.01); n.s., not significant (*P* > 0.05).

To explore the spleen’s involvement in the CAIP mechanism, mice underwent splenectomy before sepsis induction, followed by treatment with BTO@Cap/+US (Figure 8a).^68,69^ As shown in Figure 8b, the reduction in pro-inflammatory cytokines observed in septic mice treated with BTO@Cap/+US was absent in those without a spleen. This finding suggests that the anti-inflammatory effects of the treatment were dependent on the spleen, indicating its crucial role as a key target in the CAIP mechanism.

It is well-known that reserpine depletes catecholamines, including NE, in various organs and tissues, thus inhibiting sympathetic transmission.^11,23^ To delve deeper into its anti-inflammatory mechanism, mice with LPS-induced sepsis were given reserpine pretreatment to deplete NE before receiving VNS via BTO@Cap/+US (Figure 8c). Healthy mice and LPS-induced sepsis mice without reserpine pretreatment served as control groups. Compared to healthy mice, those with LPS-induced sepsis exhibited significantly lower splenic NE and ACh concentrations (Figure 8d,e), resulting in higher serum TNF-α concentration (Figure 8f). VNS activated by BTO@Cap/+US notably increased NE and ACh productions in the spleen of LPS-induced sepsis mice without reserpine pretreatment, consequently inhibiting serum TNF-α concentration. Conversely, in mice pretreated with reserpine, VNS failed to raise splenic NE and ACh productions or reduce systemic TNF-α levels. These findings suggest that BTO@Cap/+US-induced VNS enhances splenic NE and ACh productions, thereby reducing TNF-α production in specific spleen macrophages through functional signaling along catecholaminergic nerve fibers in the splenic nerve.

To determine the roles of ACh-producing ChAT^+^ T-cells and α7 nAChR expressed on macrophages, spleens from septic mice treated with BTO@Cap/+US were collected for analysis. Immunofluorescence staining was performed to visualize ChAT markers and α7 nAChR expression. For comparison, spleens from untreated septic mice and healthy controls were also analyzed. As shown in Figure S13a,b, the relative expressions of ChAT and α7 nAChR were higher in the BTO@Cap/+US group compared to those observed in the untreated control group. Similar results from traditional invasive VNS have been reported in the literature.^70,71^ These findings correspond with the elevated levels of NE and ACh observed in the spleens of BTO@Cap/+US-treated mice compared to untreated mice (Figure 8d,e). These results provide deeper insights into the cellular mechanisms underlying CAIP, supporting the observed therapeutic effects.

The BTO@Cap particles are specifically designed to target the gastric surface to stimulate the vagus nerve noninvasively and are excreted from the body without systemic absorption. This design ensures that the proposed therapy operates independently of antibiotics, minimizing the risk of potential interactions while allowing for effective bacterial clearance. Combining this system with antibiotics is a promising dual-action strategy to mitigate inflammation and address the bacterial source. However, further studies are necessary to explore possible interaction effects and optimize this approach for clinical application.

## Conclusions

The above findings suggest that the proposed noninvasive electrical VNS, activated by BTO@Cap/+US, effectively transmits stimulation signals to the brain and regulates a neural-immune network via the CAIP. This results in a reduction of systemic inflammation and major organ inflammation in sepsis mice, leading to mitigation of weight loss and death by modulating immune cells in the spleen. The activation of orally ingested BTO@Cap particles, facilitated by portable low-intensity pulsed US equipment, results in minimal thermal effects, ensuring the safety and effectiveness of the therapy. This combination of orally ingestible BTO@Cap particles and portable US equipment facilitates home-based neuroimmunomodulation therapy for treating sepsis patients, warranting accessibility and effectiveness irrespective of medical staff availability. Additionally, this noninvasive electrical VNS for immune modulation may have prospective applications in treating various cytokine-mediated diseases. This highlights the broader therapeutic potential of this approach beyond sepsis treatment.

## Supporting Information Available

Experimental section, TEM image, current–time curves, local levels of ROS, CLSM images, NIR-II images, infrared thermographs, open field test data, long-term therapeutic study data, fMRI data, and serum levels of pro-inflammatory cytokines (PDF)
