# Supplementary material for: Noninvasive Vagus Nerve Electrical Stimulation for Immune Modulation in Sepsis Therapy
Source: J Am Chem Soc. 2025 Mar 4;147(10):8406–21. doi: 10.1021/jacs.4c16367 (PMC11912339; doi:10.1021/jacs.4c16367)
Supplement: Supplementary file 1 — ja4c16367_si_001.pdf [file ja4c16367_si_001.pdf]

Supporting Information

Noninvasive Vagus Nerve Electrical Stimulation for Immune Modulation in Sepsis Therapy

Cam-Hoa Mac[1,†], Giang Le Thi Nguyen[1,†], Dien Thi My Nguyen[1,†], Sheng-Min Huang[2], Hsu-Hsia Peng[3], Yen Chang[4], Shih-Kai Lo[1], Hui-Hua Kenny Chiang[5], Yuan-Zhen Yang[5], Hsiang-Lin Song[6], Wei-Tso Chia[7], Yu-Jung Lin[8,*], Hsing-Wen Sung[1,*]*

[1] Department of Chemical Engineering, National Tsing Hua University, Hsinchu, Taiwan

[2] Department of Pharmacology, College of Medicine, National Cheng Kung University, Tainan, Taiwan

[3] Department of Biomedical Engineering and Environmental Sciences, National Tsing Hua University, Hsinchu, Taiwan

[4] Taipei Tzu Chi Hospital, Buddhist Tzu Chi Medical Foundation and School of Medicine, Tzu Chi University, Hualien, Taiwan

[5] Institute of Biomedical Engineering, National Yang-Ming Chiao Tung University, Taipei, Taiwan

[6] Department of Pathology, National Taiwan University Hospital, Hsinchu Branch, Hsinchu, Taiwan

[7] Department of Orthopedics, National Taiwan University Hospital, Hsinchu Branch, Hsinchu, Taiwan

[8] Research Center for Applied Sciences, Academia Sinica, Taipei, Taiwan

Keywords: sepsis, piezoelectric material, vagus nerve stimulation, neuroimmunomodulation, cholinergic anti-inflammatory pathway

Experimental section

Materials. Tetrabutyl orthotitanate (TBOT) and barium hydroxide (BH) were acquired from Thermo Fisher Scientific (Fair Lawn, NJ, USA), while Cap was obtained from Tokyo Chemical Industry (Tokyo, Japan). Hydrogen peroxide (H_2O_2, 30 wt%), 3-mercaptopropyltriethoxysilane (MPTES), Irgacure® 2959, polyoxyethylene (23) lauryl ether, anhydrous tetrahydrofuran (THF), (3-aminopropyl)-triethoxysilane (APTES), and CPZ were procured from Sigma-Aldrich (St. Louis, MO, USA). Additionally, ICG NHS ester (ICG-OSu) was purchased from Med Chem Express (Monmouth Junction, NJ, USA), terephthalic acid from Alfa Aesar (Heysham, England), and XTT (sodium salt hydrate) from Cayman Chemical (Ann Arbor, MI, USA). Caco-2 cells and SH-SY5Y cells were obtained from the American Type Culture Collection (Manassas, VA, USA). All chemicals and reagents used were of analytical grade.

BTO particle synthesis. In the study, 45 mL of ethanol was mixed with 0.18 mL of a 0.1 M solution of aqueous polyoxyethylene (23) lauryl ether. Subsequently, 1.36 mL of TBOT (4 mmol) was added under a nitrogen gas atmosphere at room temperature with stirring using a magnetic stirrer. Stirring ceased after 18 min. After 24 h, the reaction concluded, and the resulting spheres were collected via centrifugation and rinsed with ethanol. The prepared TiO_2 spheres were dispersed in 40 mL of deionized (DI) water and divided into 4 tubes (10 mL/tube). A 10 mL solution of dispersed TiO_2 was added to a 40 mL solution of BH (2 mmol) and sonicated at room temperature for 60 min. The final solution was transferred to a Teflon-lined stainless-steel autoclave and heat-treated at 220°C for 24 h. The synthesized BTO particles were subsequently centrifuged at 5,000 rpm for 3 min and washed four times using DI water/acetic acid (0.05 wt%)/ethanol, followed by rinsing with DI water.

BTO@Cap particle synthesis. Initially, 350 mg of the synthesized BTO particles were refluxed with a 200 mL aqueous solution of H_2O_2 (30 wt%) at 105°C for 16 h with stirring. The resulting

BTO–OH particles were collected, washed with DI water and ethanol, and dried overnight in an oven. Subsequently, Cap-MPTES was synthesized via the thiol–ene click reaction.[1,2] Briefly, MPTES (130 μL), Cap (164 mg), and the photoinitiator (Irgacure®2959, 3.6 mg) were mixed in 2 mL of anhydrous THF. The reaction mixture was subjected to UV light for 48 h under nitrogen gas with gentle stirring to obtain Cap-MPTES. Finally, the solvent was removed from the synthesized Cap-MPTES using a rotary evaporator. Cap-MPTES was then reacted with BTO–OH (80 mg) in toluene solvent (5 mL) under a nitrogen gas atmosphere at 40°C for 24 h. The resulting BTO@Cap particles were collected, washed with toluene and ethanol, and dried overnight in an oven at 40°C. The non-piezoelectric cubic BTO particles were synthesized using a similar procedure as tetragonal BTO particles, with modifications to the reaction temperature and duration, adjusted to 180°C and 10 h, respectively.[3,4]

Characterization of BTO@Cap particles. The morphological structures of the synthesized BTO and BTO@Cap particles were examined using SEM (JSM-5600, JEOL Technics, Tokyo, Japan) and TEM (JEM-F200, JEOL Technics, Tokyo, Japan). The crystalline structure and phase composition of both particles were determined using Micro Raman Identify Dual system (MRID-Raman, ProTrusTech Co., Ltd., Tainan, Taiwan) and XRD (Cu Kα radiation, XRD-6000, Shimadzu, Tokyo, Japan). The loading content of Cap on the BTO@Cap particles was analyzed via TGA (Pyris Diamond, Perkin Elmer, USA), measuring the weight loss compared to BTO–OH particles to determine the amount of Cap loaded.

Piezoelectric properties of test particles. The piezoelectric properties of BTO and BTO@Cap particles were assessed using PFM (Cypher S AFM Microscope, Oxford Instruments-Asylum Research, Santa Barbara, USA).

To demonstrate the ability of BTO and BTO@Cap particles to generate current under US activation, a lighting experiment was conducted. A device comprising a thin piezoelectric film

containing the particles sandwiched between two flexible platinum electrodes was prepared. The AC output voltage collected from particles subjected to US exposure was converted into DC output using a full-wave rectifier built with a diode (1N4002). This DC output was then used to charge a capacitor (1 μF) (Figure S2a). Finally, the charged capacitor powered an LED bulb.[5,6] The output current of test particles was measured in the LED lighting experiment using a Keithley 2400 instrument (Keithley Instruments, Inc., Cleveland, OH, USA) to capture the current signal after the capacitor had been discharged.

To further confirm current generation under conditions that better simulate in vivo environments, an experiment was conducted as outlined in Figure S2b. In this setup, BTO or BTO@Cap particles were coated onto FTO glass connected to a measurement device.[7,8] The FTO glass served as the working electrode, with a platinum counter electrode and an Ag/AgCl reference electrode.[7,8] The coated particles were immersed in an electrolyte solution (0.1 M NaCl) to simulate body fluids, while US waves were applied from approximately 1 cm away, outside the glass wall of the beaker. The current-time (I–t) responses of the samples, under US irradiation at a frequency of 1.0 MHz, intensity of 0.3 W/cm^2, and a 50% duty cycle, were recorded using an ELITE EDC wireless data acquisition system (Biopro Scientific, Hsinchu, Taiwan). To ensure efficient transmission of US waves through the glass wall and into the solution, US conductive gel was applied to the probe in all experiments.

Finite element modeling (FEM) simulation. The piezoelectric potential simulation was performed using the finite element method (FEM) in COMSOL Multiphysics software (Burlington, MA, USA). The simulation model consisted of a BTO particle with a 1 μm radius, surrounded by a water medium with infinite boundaries (relative permittivity, ε_r = 80). To analyze the piezoelectric properties, a continuous surface pressure of 7×10^4 Pa was applied to the particle. The ultrasonic vibration in the surrounding water had a frequency of 1 MHz. The other material

parameters used in this simulation, including the elasticity matrix (cE) of BTO particle, were obtained from the predefined material properties available in COMSOL Multiphysics. The polarization of the BTO particle is aligned along the z-axis of the global coordinate system, with its center fixed and grounded. Continuous ultrasound was applied to study the surface pressure on the particle. The result shown in Figure 2c captures the electron distribution at the precise moment of 1.2 ns, representing our current computational capacity.

Stability of test particles under GI conditions. To assess the stability of BTO and BTO@Cap particles in the GI environment under US stimulation, the particles were individually incubated in SGF and SIF. The incubation involved subjecting the particles to SGF for 2 h or SIF for 3 h at 37°C with continuous shaking. SGF and SIF were prepared according to established protocols.[1,9] Specifically, SGF comprised an HCl solution with a pH of 2.0 and a pepsin concentration of 0.5 mg/mL (Sigma-Aldrich), while SIF was a neutral solution with a pH of 7.0 containing 5 mg/mL bile extract and 1.6 mg/mL lipase (Sigma-Aldrich). TGA was utilized to quantify the Cap content grafted onto the surfaces of BTO@Cap particles before and after exposure to SGF or SIF. Additionally, SEM was employed to examine the morphology and size of the collected test particles.

Assessment of ROS production levels upon US stimulation. ROS production levels were examined upon exposing BTO@Cap particles to varying durations and power intensities of US stimulation. The investigation focused on assessing the levels of H_2O_2, •OH, and $•O_2^-$ in PBS. H_2O_2 production was quantified using the Amplex™ Red Hydrogen Peroxide/Peroxidase Assay Kit (Invitrogen Thermo Fisher Scientific, Eugene, OR, USA). Additionally, the production of •OH and $•O_2^-$ was evaluated using TA and XTT as indicators, respectively.

Cell culture conditions. The Caco-2 cells were cultured in Minimum Essential Medium (MEM, Gibco, Grand Island, NY, USA) supplemented with 10% fetal bovine serum (FBS, Gibco), 1%

MEM non-essential amino acids (Gibco), 1% sodium pyruvate solution (Biological Industries, Kibbutz Beit Haemek, Israel), and 1% penicillin/streptomycin (Gibco). On the other hand, the SH-SY5Y cells were maintained in Dulbecco's Modified Eagle's medium/F-12 (DMEM/F-12, Gibco) supplemented with 10% FBS and 1% penicillin/streptomycin. Both cell types were incubated in T75 flasks at 37°C in a humidified atmosphere with 5% CO_2.

Cytotoxicity study. Caco-2 cells (1×10^5 cells per well) were seeded in 24-well plates and allowed to attach for 24 h. Following attachment, the cells were treated with BTO@Cap particles at various concentrations ranging from 0 to 5 mg/mL. Subsequently, the treated cells were exposed to US irradiation at different power intensities ranging from 0 to 0.5 W/cm^2 (1 MHz and 50% duty cycle). After an additional 24-h incubation period, cell viability was assessed using the CellTiter-Glo® Luminescent Cell Viability Assay Kit (Promega, Madison, Wisconsin, USA).

BTO@Cap/+US efficacy in VNS initiation. The efficacy of BTO@Cap/+US as piezoelectric stimulators for initiating VNS was investigated in vitro using a transwell culture model. Initially, the upper chamber of a 24-well transwell was seeded with Caco-2 cells (1×10^5 cells per well), while the lower chamber of another well plate was seeded with SH-SY5Y cells (1×10^5 cells per well). After three days of incubation, the upper chamber was treated with BTO@Cap particles (1 mg/mL) and cultured for 2 h. To verify the targeting of particles to TRPV1, BTO@Cap particles were co-administered with CPZ (7 μM), a selective blocker of TRPV1.[10] During this time, SH-SY5Y cells in the lower chamber were treated with a calcium ion (Ca^{2+}) indicator (Fluo-8, Abcam, Cambridge, UK, 5 μM) for 30 min in darkness at 37°C. Subsequently, the SH-SY5Y cells in the lower chamber were washed with PBS and supplied with artificial cerebrospinal fluid (TOCRIS, Ellisville, MI, USA).[11] The Caco-2 cells in the upper chambers were also washed with PBS and then co-cultured with the SH-SY5Y cells in lower chamber, and exposed to US activation (1 MHz, 0.3 W/cm^2, 50% duty cycle, 3 doses). Following US exposure, fluorescence images were captured

using CLSM.

Animal study. Male C57BL/6 mice, aged six to eight weeks, were sourced from the National Laboratory Animal Center in Taipei, Taiwan. All animal experiments followed the guidelines outlined in the "Guide for the Care and Use of Laboratory Animals" prepared by the Institute of Laboratory Animal Resources, National Research Council, and published by the National Academy Press in 2011. Approval for the animal research protocols was obtained from the Institutional Animal Care and Use Committee of National Tsing Hua University (Approval number: 112059).

Biodistribution study. In the biodistribution study, mice were exclusively fed a low-fluorescent diet for three days before the experiment and subjected to a 12-h fasting period. They were then divided into different groups: untreated and groups receiving oral administration of ICG-labeled BTO and ICG-labeled BTO@Cap particles (40 mg/kg). To prepare the fluorescently labeled particles, 80 mg of either BTO–OH or BTO@Cap particles were suspended in 5 mL of toluene. Following this, 1 mL of APTES was added to the suspension, and the mixture underwent a 24-h reaction at 40°C under a nitrogen atmosphere.[12] Subsequently, the resulting particles, terminated with amines, were covalently conjugated with ICG NHS ester, following the manufacturer's instructions. To remove any unconjugated fluorescence molecules, the particles underwent multiple washes using DI water and centrifugation.

The stability of the ICG label on particles under the strongly acidic conditions of gastric juice was assessed through an in vitro study evaluating the NIR signal retention of fluorescently labeled BTO (f-BTO) particles. In this experiment, ICG-labeled BTO particles were immersed in SGF, thoroughly mixed, and incubated for 2 h. After incubation, the particles were centrifuged to separate the solid particles from the SGF solution, and the NIR signal was analyzed using an imaging system (ZephIR™ 1.7, Photon etc., Montreal, Quebec, Canada). As a control, the NIR

signal was also captured immediately after adding ICG-labeled-BTO particles to SGF and allowing them to settle.

At predetermined time intervals (10, 20, 30, 40, 50, and 90 min) after treatment, the distribution of accumulated particles in the stomach was observed using an NIR camera. In the inhibitor group, test mice were intraperitoneally injected with CPZ (5 mg/kg) before the oral administration of ICG-labeled BTO@Cap particles.[13] Additionally, the distribution of accumulated ICG-labeled BTO@Cap particles in major visceral organs, including the brain, heart, lung, liver, spleen, pancreas, kidneys, stomach, and intestine, was observed at longer predetermined time intervals (4, 6, 8, 12, and 24 h) after treatment. The excretion of ICG-labeled BTO@Cap particles through collected feces was also examined at various time intervals using the NIR camera.

Cell Attachment Test. BTO and BTO@Cap particles pre-labeled with Alexa Fluor 633 (f-BTO and f-BTO@Cap particles) were synthesized by covalently conjugating Alexa Fluor 633 NHS ester (Thermo Fisher Scientific, Waltham, MA, USA) to amine-terminated particles, following the manufacturer's protocol. Unbound fluorescent molecules were removed by repeated washing and centrifugation with DI water. To evaluate targeted attachment, Caco-2 cells were incubated with f-BTO@Cap particles, with untreated cells and cells treated with f-BTO particles serving as controls. Caco-2 cells were seeded in 8-well chamber slides (2.5×10^4 cells/well) and incubated for 24 h to ensure attachment. Cells were then treated with f-BTO@Cap or f-BTO particles (10 μg/mL) and incubated. Afterward, the medium was aspirated, and cells were washed three times with DPBS to remove unbound particles. Cells were fixed with 4% paraformaldehyde for 15 min at room temperature, followed by staining the cell membranes with DiI (20 μg/mL, Thermo Fisher Scientific) and nuclei with Hoechst 33342 (1:1000 dilution, Abcam, Cambridge, MA, USA). CLSM was used to visualize the attachment of particles on the surface of Caco-2 cells.

Biosafety assessment. Blood samples were collected from healthy mice, both with and without

treatment of BTO@Cap particles (40 mg/kg) and 3 doses of US activation. These samples were utilized for AST, ALT, BUN, and CRE tests. Subsequently, the mice were sacrificed, and all major organs were harvested for histological staining (hematoxylin–eosin, H&E).

Open field test. The locomotor activities of test mice were assessed using an open field test with live-video tracking (EthoVision XT, Noldus Information Technology Inc., Leesburg, VA, USA). The total distance traveled by each mouse during the test was recorded for subsequent analysis.

Therapeutic efficacy. The investigation of the therapeutic efficacy of combining oral BTO@Cap particles with US in a three-dose treatment was conducted using a sepsis mouse model. For the survival study, mice were given intraperitoneal injections of LPS (20 mg/kg) to induce sepsis[14] (Figure 5a). After 10 min, the mice were orally administered DI water in the untreated and US groups, BTO@Cap particles (40 mg/kg) in the BTO@Cap and BTO@Cap/+US groups, or BTO particles in the BTO and BTO/+US groups. Following an additional 20 min, the mice in the US, BTO/+US, and BTO@Cap/+US groups were subjected to US exposure (1 MHz, 0.3 W/cm^2, 50% duty cycle, 3 doses), with the US transducer applied externally to the stomach area. Survival and body weight were then monitored for 7 days. To further investigate the long-term effects of BTO@Cap/+US treatment, the survival rate and body weight of the mice were monitored for up to 21 days post-treatment. The first seven days of this extended study are reflected in the survival and body weight data presented in Figures 5b and 5c, with the sample size increased to 12–15 mice per group.

For the remainder of the therapeutic efficacy study, mice were intraperitoneally injected with LPS (10 mg/kg).[15] The treatment procedure and timeline followed the same protocol as the survival rate and body weight study (Figure 6a). To determine the concentrations of inflammatory cytokines, blood samples were individually collected from mice at 1 h, 6 h, and 10 h post-LPS injection. The levels of pro-inflammatory cytokines (TNF-α, IL-1β, IL-17A, IL-6, and IFN-γ) in

the serum were assessed using a Multi-Plex Immunoassay (Bio-Plex Pro, Bio-Rad Laboratories, Hercules, USA). Additionally, serum analysis was performed to evaluate hepatic and renal functions by assessing ALT, AST, CRE, and BUN levels. Following the third blood collection, all mice were euthanized, and their organs were collected, fixed in 4% formaldehyde, embedded in paraffin, and sectioned for H&E staining.

Immunofluorescence staining of NTS. For the investigation of their neural activity, sepsis mice subjected to BTO@Cap treatment (40 mg/kg) with three doses of US activation were sacrificed immediately after the treatment. The brains were collected and fixed in 4% paraformaldehyde at 4°C before being embedded in paraffin. Untreated sepsis mice were used as a control. For the vagotomy group, mice were anesthetized intramuscularly with a mixture of Zoletil 50 (25 mg/mL, 20 μL), Rompun (20 mg/mL, 5 μL), and DPBS (75 μL). Both the left and right cervical vagal nerve trunks were transected prior to sepsis induction, followed by the administration of BTO@Cap/+US treatment or no treatment.[16] Brain tissue sections were deparaffinized, followed by incubation with anti-c-Fos antibody (1:1000, Abcam ab222699), and then treated with Alexa Fluor 633 Dye Goat Anti-Rabbit Antibody (1:200, Thermo Fisher Scientific) as a secondary antibody. Finally, the sections were counterstained with DAPI (1:1000, Thermo Fisher Scientific), and the stained samples were examined using CLSM.

MRI acquisition. The investigation of the regions where the forebrain was activated by the BTO@Cap/+US in mice was conducted using rs-fMRI. The timeline and experimental setup for the MRI study was illustrated in Figure S14. MRI data were collected using a 7 T animal scanner (Bruker Biospec 70/20 USR, Germany) equipped with a mouse brain surface radio-frequency coil for acquisition. During MRI scans, the mice were anesthetized using dexmedetomidine (administered at an initial dose of 0.025 mg/kg followed by a continuous infusion of 0.05 mg/kg/h via intraperitoneal injection) along with a mixture of 0.5% isoflurane/oxygen (1 L/min). The mice

were secured in a bed with warm water circulation system, and the respiratory rate was monitored using a pressure sensor (SA Instruments, Inc., NY, USA), ranging from 70 to 160 respirations per minute during MRI scans.

The brain functional images were acquired in transversal view and 16 consecutive slices were obtained by using a gradient-echo echo planar imaging sequence. The magnetic field homogeneity was refined with Bruker FieldMap shim process. The scanning parameters for rs-fMRI were field-of-view (FOV) of 20 × 20 mm^2, matrix size of 80 × 80, slice thickness of 0.5 mm, repetition time (TR) of 2,000 ms, echo time (TE) of 21 ms, and bandwidth of 500 kHz. Two rs-fMRI sessions were acquired in each mouse and each session contained 300 volumes of images. Each session took 10 min for acquisition. Structural T2-weighted images (T2WI) were acquired using turbo spin-echo acquisition with identical slice geometry to rs-fMRI images. The scanning parameters of T2WI were FOV of 20 × 20 mm^2, matrix size of 256 × 256, slice thickness of 0.5 mm, TR of 2,500 ms, effective TE of 40 ms, turbo factor of 8, and 4 averages.

MRI data processing and analysis. Preprocessing on the rs-fMRI data was conducted using the open-source RABIES software, available at https://github.com/CoBrALab/RABIES),[17] following the standard pre-processing steps outlined by Grandjean et al.[18] Specifically, the first 10 volumes of rs-fMRI data were excluded as MRI signals had not yet reached a steady state. Head motion parameters were estimated across Echo Planar Image (EPI) frames using a rigid-body registration. For each mouse, the volumetric EPI underwent field inhomogeneity correction to address EPI susceptibility distortions, and was then normalized to its structural T2WI via nonlinear registration. To align with a common space, each mouse's structural T2WI was first corrected for inhomogeneities and then individually aligned to the Dorr-Steadman-Ullmann-Richards-Qiu-Egan (DSURQE) mouse brain atlas, accessible at https://wiki.mouseimaging.ca/display/MICePub/Mouse+Brain+Atlases, built into the RABIES

software using nonlinear registration.[19–23]

Spatial transformations for motion correction, susceptibility distortion correction, and common space normalization were combined into a single resampling, which was then applied to each EPI frame to generate EPI in a common space. A voxel resolution of 0.1 × 0.1 × 0.5 mm^3 was used for spatial resampling. Following spatial processing, confound correction for nuisance regressors, including linear trend, motion parameters, and mean signal from white matter and cerebrospinal fluid, was performed. A bandpass-filter of 0.01–0.1 Hz was applied. A spatial Gaussian smoothing filter with 0.4 mm full-width at half maximum (FWHM) was then applied. The 3dRSFC function in AFNI toolbox, available at http://afni.nimh.nih.gov, was employed to calculate the fALFF maps. For regional analysis, 30 targeted brain regions in common space were selected from the DSURQE atlas (Figure 7c), and the regional fALFF values were extracted. The bandpass-filtered time series was used to calculate the functional connectivity among brain regions. Specifically, voxel-wise rs-fMRI signals within each region were averaged. Pearson's correlation coefficient (r) of rs-fMRI signals between two regions was computed to indicate the functional connectivity between the selected brain regions, generating a 30 × 30 correlation matrix for each mouse. To assess the centrality (D) of brain hub i, the degree centrality of each region was estimated after binarizing the 30 × 30 correlation matrix with a threshold of $r > 0.2$ and using the following expression: $D^i = \sum a_{ij}\ where\ j = 1 \ldots 30, i \neq j.$ a_{ij} is the connection status (the value in the binarized matrix) between nodes i and j.

Splenectomy. To investigate the significant role of the spleen as a target organ in the treatment mechanism of CAIP, mice were anesthetized with an intramuscular injection of a mixture containing Zoletil 50 (25 mg/mL, 20 µL), Rompun (20 mg/mL, 5 µL), and DPBS (75 µL). The splenic arteries were ligated using sutures, after which the spleen was extracted.[24,25] Following the surgery, the animals were allowed to recover for 7 days before undergoing LPS induction and

BTO@Cap/+US treatment (Figure 8a). Control groups included mice that underwent splenectomy without any treatment and those that underwent splenectomy with LPS induction but without BTO@Cap/+US treatment.

Exploring CAIP in treatment mechanisms. To investigate whether the treatment mechanism followed the CAIP, a mechanism study was conducted (Figure 8c). In this study, untreated mice received an intraperitoneal injection of LPS (10 mg/kg). After 10 min, mice were orally administered treatments of BTO@Cap particles (40 mg/kg). Followed by another 20 min, the mice were exposed to three doses of US.

One hour after LPS injection, the mice were anesthetized, and their spleens were collected and immediately homogenized in PBS containing a Phosphatase and Protease Inhibitor Cocktail (1X, Celltechgen, Houston, USA). ACh and NE levels were measured using the Choline/Acetylcholine Assay kit (Abcam) and Noradrenaline High Sensitive ELISA kit (LDN, Nordhorn, Germany), respectively, while serum TNF-α levels were determined using a Multi-Plex Immunoassay.

Additionally, some spleens were immediately fixed in 4% paraformaldehyde at 4°C overnight, dehydrated, embedded in O.C.T. Compound (Sakura Finetek, Torrance, CA, USA), and stored at −80°C for cryosectioning. The samples were immunofluorescently stained with Rabbit Polyclonal Nicotinic Acetylcholine Receptor alpha 7 antibody (Ab216485, Abcam, 1:200 dilution) or Rabbit Recombinant Monoclonal Choline Acetyltransferase antibody (Ab178850, Abcam, 1:200 dilution). Afterward, the samples were incubated with the secondary antibody (Alexa Fluor 488 Anti-Rabbit Antibody, A11070, Thermo Fisher Scientific, 1:200 dilution). Finally, the sections were counterstained with DAPI (Thermo Fisher Scientific, 1:1000 dilution) and examined using CLSM.

To further explore the role of NE in this pathway, catecholamine stores were depleted by treating mice with reserpine before conducting the mechanistic study.[26] A dose of 10 mg/kg reserpine was

intraperitoneally administered to mice 24 h before the beginning of the study. Afterward, the mice were anesthetized, and their spleens were collected and immediately homogenized to measure splenic ACh and NE levels. Additionally, serum TNF-α levels were measured to assess the inflammatory response.

Statistical analysis. All quantitative data are presented as mean ± standard deviation. Statistical analysis was conducted using the two-tailed Student's *t*-test to compare independent groups, with statistical significance set at $P < 0.05$ (indicated by *), $P < 0.01$ (**), and $P < 0.001$ (***).

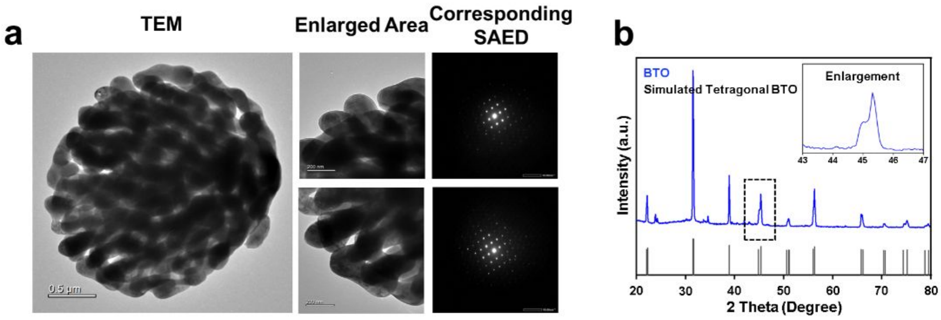

Figure S1. a) TEM image of a single BTO particle, including magnified views of selected regions and corresponding SAED pattern. b) XRD pattern of BTO particles compared to the simulated pattern for tetragonal BTO.

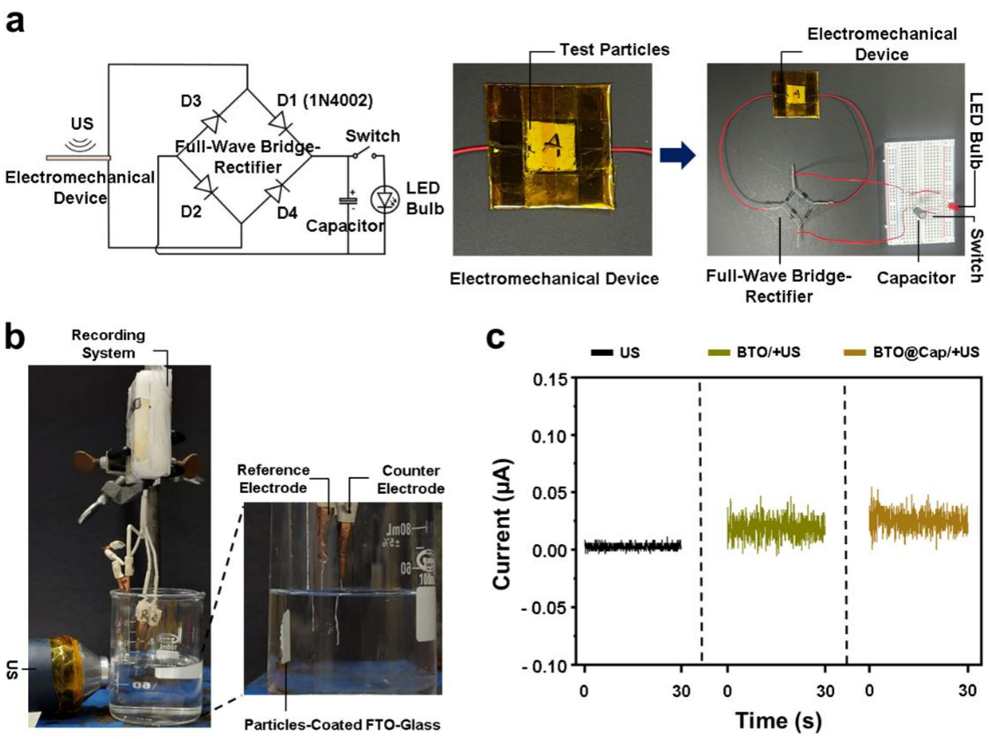

Figure S2. a) Schematic illustrations and photographs of the electromechanical measurement device used for testing LED bulb illumination and measuring piezoelectric current generated by BTO or BTO@Cap particles. b) Photograph showing the ex vivo current measurement experiment, designed to better simulate in vivo conditions. c) Current–time curves for the empty FTO substrate and the BTO- or BTO@Cap-coated FTO substrates under US stimulation.

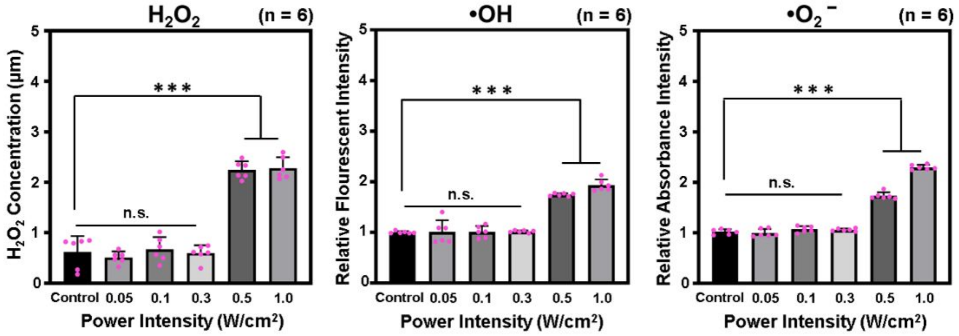

Figure S3. Local levels of H_2O_2, •OH, and $•O_2^-$ detected in PBS containing BTO@Cap particles after US stimulation at varying power intensities. Each dot represents a data point from the observed measurements. Statistical significance is indicated as follows: ***($P < 0.001$); n.s. denotes not significant ($P > 0.05$).

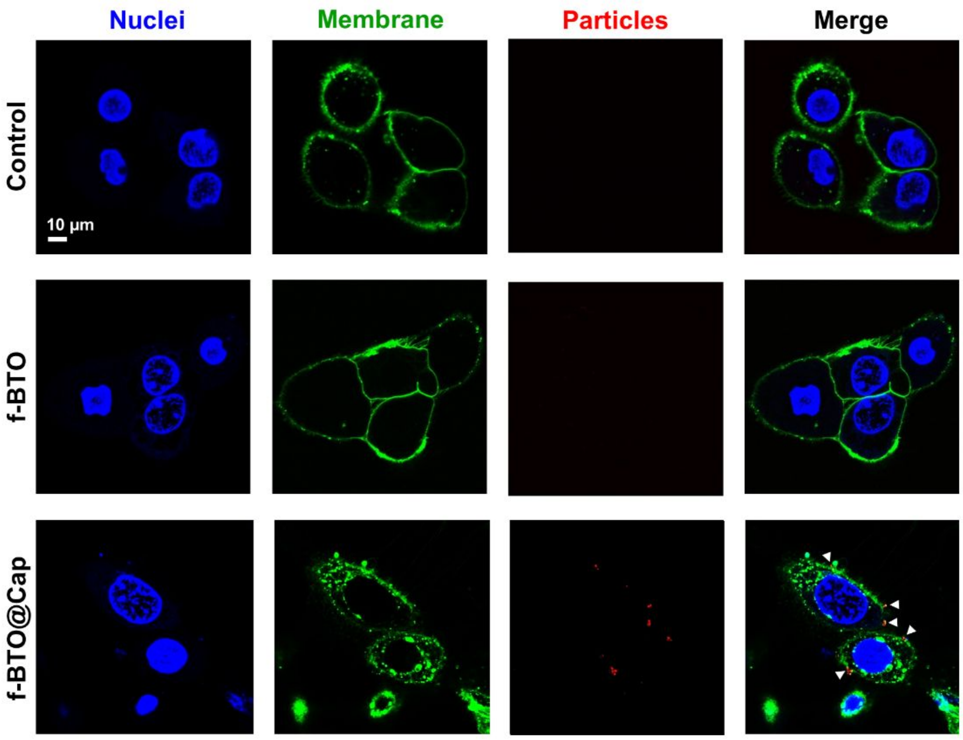

Figure S4. CLSM images showing the attachment of f-BTO@Cap particles to Caco-2 cells, with nuclei stained in blue and cell membranes stained in green. White arrowheads indicate the attachment of particles to the cell membranes.

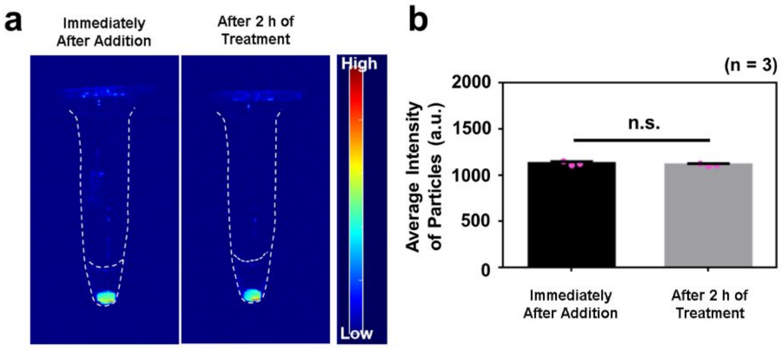

Figure S5. a) NIR-II images showing the fluorescence signal of ICG-labeled BTO particles immediately after being added to SGF and after 2 h of incubation in SGF. The dashed line outlines the Eppendorf tube and the SGF solution inside it. b) Quantitative analysis of fluorescence intensity for particles in the SGF solution, comparing the two groups. Statistical analysis indicates no significant difference (n.s., $P > 0.05$).

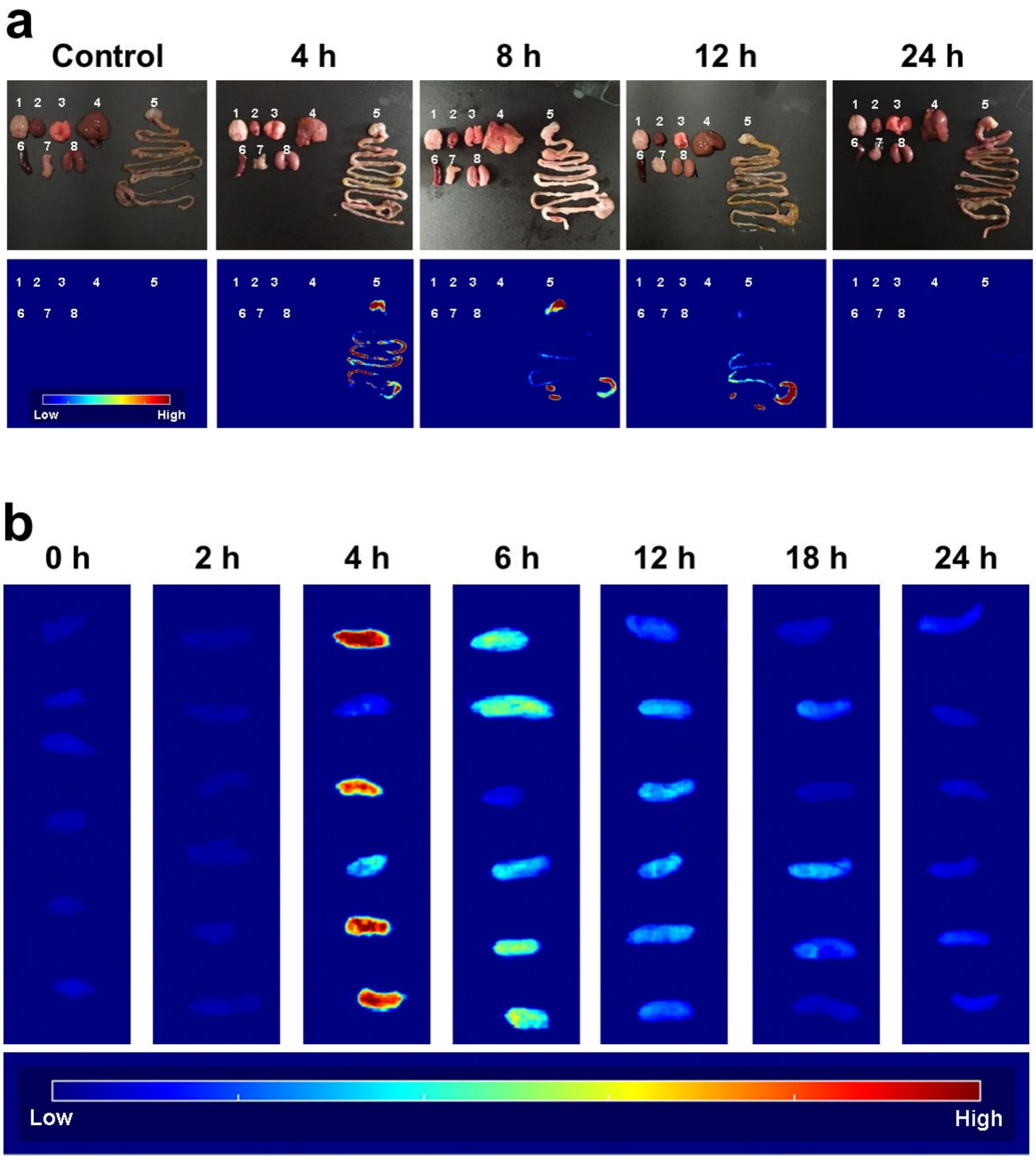

Figure S6. a) Ex vivo NIR-II images illustrating the biodistribution of ICG-labeled BTO@Cap particles after oral administration to mice. The images show no significant accumulation of particles in major visceral organs at various time points. Organs are labeled as follows: 1: brain; 2: heart; 3: lung; 4: liver; 5: gastrointestinal (GI) tract; 6: spleen; 7: pancreas; and 8: kidneys. b) Ex vivo NIR-II images displaying the biodistribution of ICG-labeled BTO@Cap particles in feces collected from test mice at different time points.

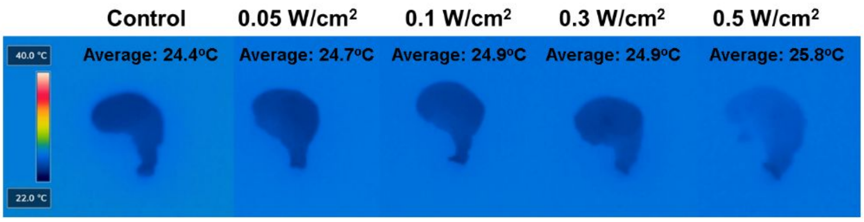

Figure S7. Infrared thermographs of stomach tissues collected from mice after oral administration of BTO@Cap particles under US irradiation at various power intensities, along with their corresponding average surface temperatures.

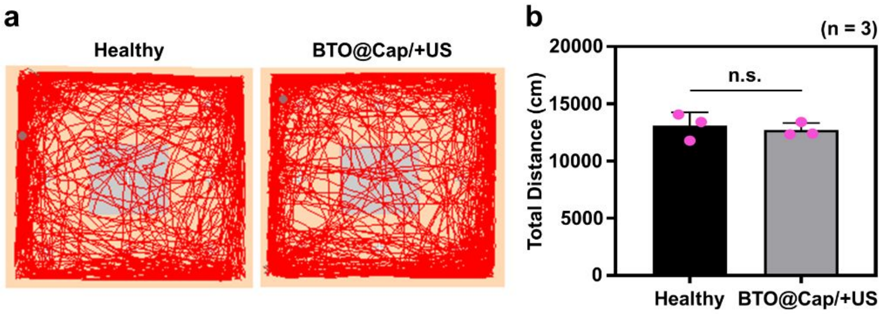

Figure S8. a) Representative trajectory plots and b) distances traveled in one hour for healthy mice and those treated with BTO@Cap/+US. n.s.: not significant ($P > 0.05$).

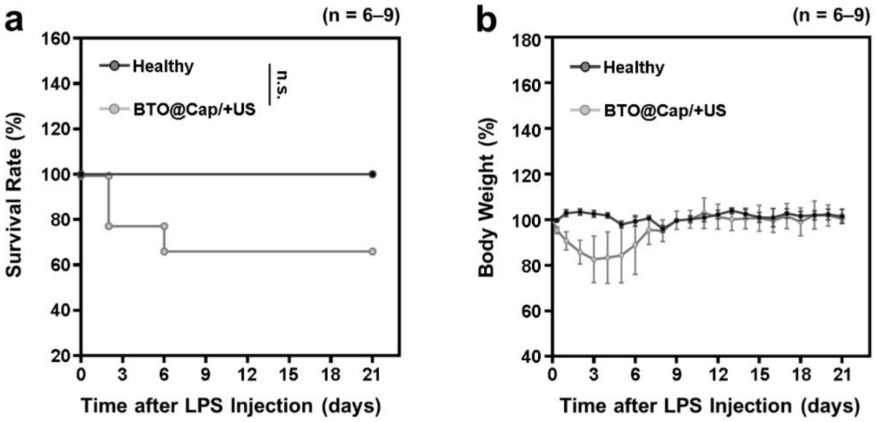

Figure S9. Long-term a) survival rate and b) body weight of healthy mice and LPS-induced septic mice after BTO@Cap/+US treatments over a 21-day period. n.s.: not significant ($P > 0.05$).

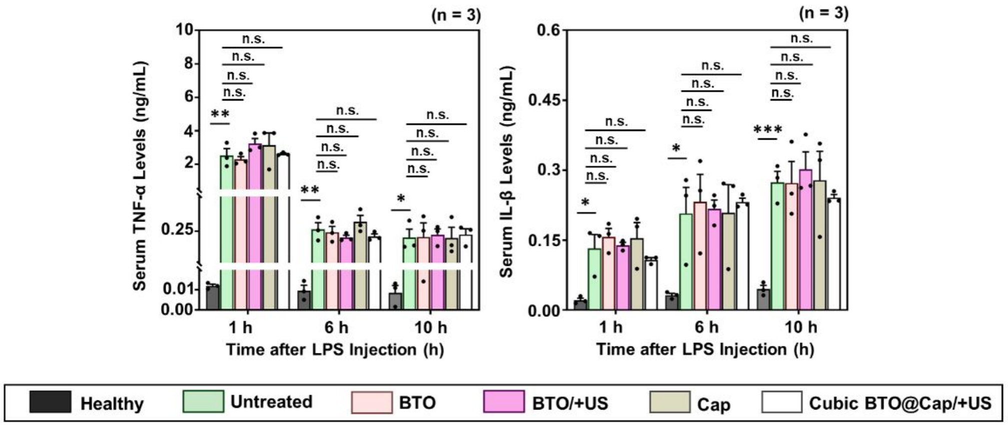

Figure S10. Serum levels of pro-inflammatory cytokines (TNF-α, and IL-1β) collected from healthy mice and septic mice after various treatments. *(P < 0.05), **(P < 0.01), ***(P < 0.001), and n.s. (not significant, P > 0.05).

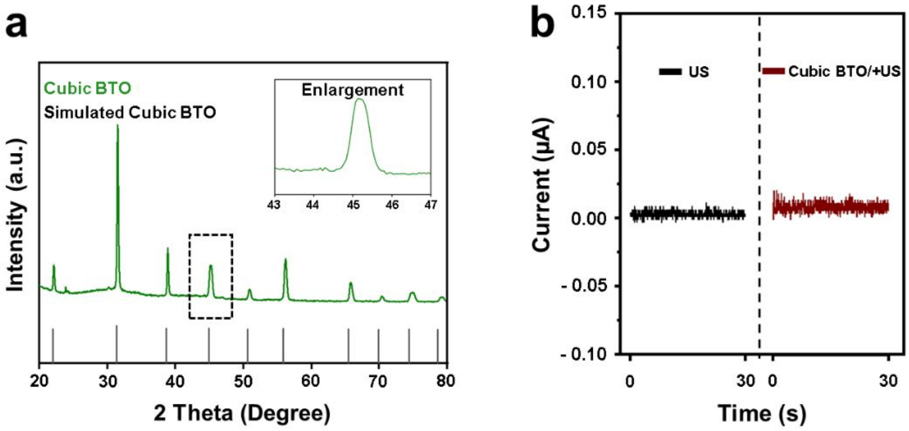

Figure S11. a) XRD pattern of cubic BTO particles, compared with the simulated pattern for cubic BTO. b) Current–time curves for the empty FTO substrate and the cubic BTO-coated FTO substrates under US stimulation.

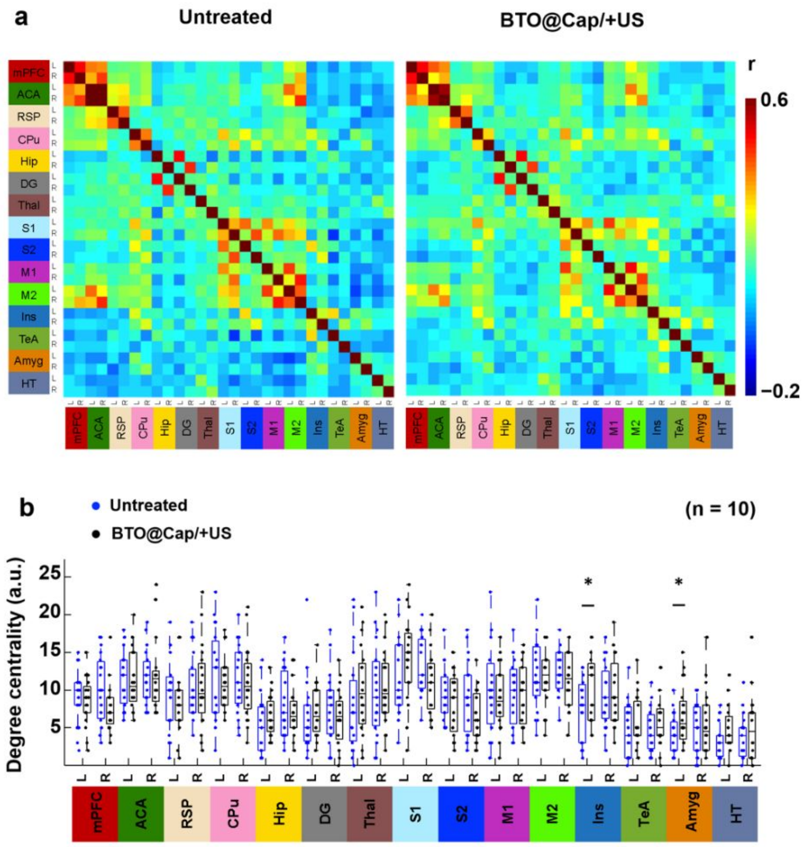

Figure S12. a) Averaged correlation matrices of the two mouse groups. b) Results of degree centrality analysis in the selected brain regions for both mouse groups. mPFC: medial prefrontal cortex; ACA: anterior cingulate area; RSP: retrosplenial area; CPu: caudate putamen; Hip: hippocampus; DG: dentate gyrus; Thal: thalamus; S1: primary so-matosensory cortex; S2: secondary somatosensory cortex; M1: primary motor cortex; M2: secondary motor cortex; Ins: insu-lar cortex; TeA: temporal association area; Amyg: amygdala; HT: hypothalamus. Each dot represents one observed data point. *($P < 0.05$).

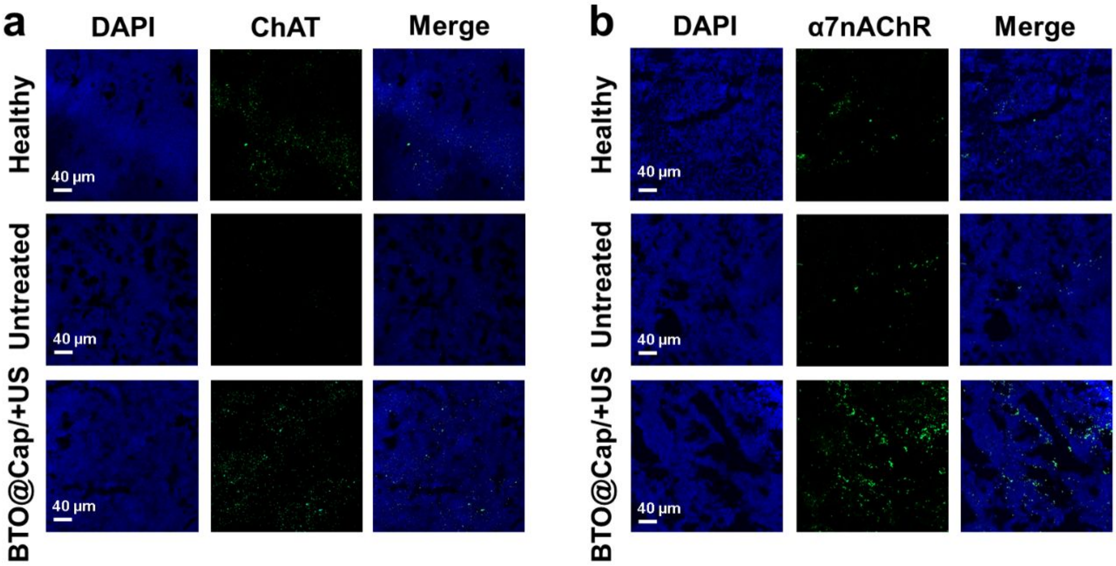

Figure S13. Immunofluorescent staining was used to detect a) ChAT (green) expression and b) α7nAChR (green) expression in the spleens of healthy mice, as well as septic mice with and without treatment.

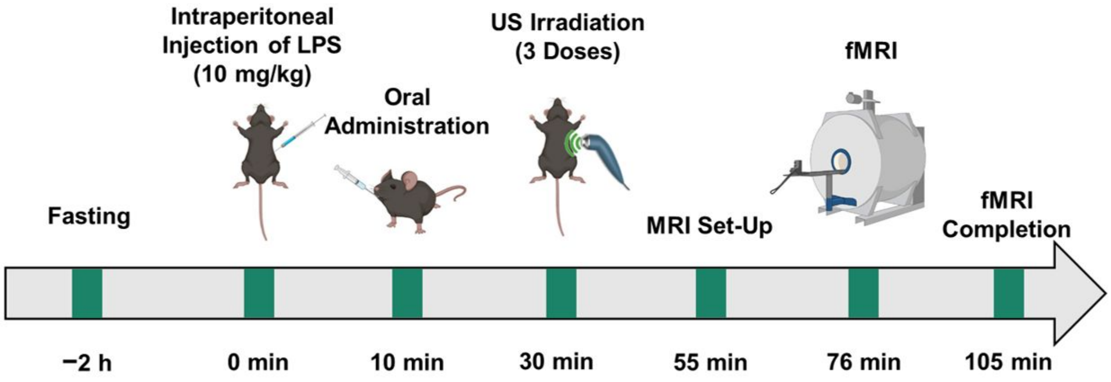

Figure S14. Schematic timeline and treatment protocol illustrating the rs-fMRI procedure used to investigate the anti-inflammatory mechanism in the brain in LPS-induced septic mice (created with Biorender.com).

References

1. Mac, C. H.; Tai, H. M.; Huang, S. M.; Peng, H. H.; Sharma, A. K.; Nguyen, G. L. T.; Chang, P. J.; Wang, J. T.; Chang, Y.; Lin, Y. J. et al. Orally ingested self-powered stimulators for targeted gut-brain axis electrostimulation to treat obesity and metabolic disorders. *Adv. Mater.* **2024**, *36* (21), e2310351, DOI: 10.1002/adma.202310351
2. Tucker-Schwartz, A. K.; Farrell, R. A.; Garrell, R. L. Thiol–ene click reaction as a general route to functional trialkoxysilanes for surface coating applications. *J. Am. Chem. Soc.* **2011**, *133* (29), 11026–11029, DOI: 10.1021/ja202292q
3. Gao, J., Shi, H., Yang, J. Li, T., Zhang, R., Chen, D. Influencing factor investigation on dynamic hydrothermal growth of gapped hollow $BaTiO_3$ nanospheres. *Nanoscale Res. Lett.* **2015**, *10*, 329, DOI: 10.1186/s11671-015-1033-x
4. Li, M.; Gu, L.; Li, T.; Hao, S.; Tan, F.; Chen, D.; Zhu, D.; Xu, Y.; Sun, C.; Yang, Z. TiO_2-seeded hydrothermal growth of spherical $BaTiO_3$ nanocrystals for capacitor energy-storage application. *Crystals* **2020**, *10*, 202. DOI: 10.3390/cryst10030202
5. Jian, G., Jiao, Y., Meng, Q., Shao, H., Wang, F., Wei, Z. 3D $BaTiO_3$ flower based polymer composites exhibiting excellent piezoelectric energy harvesting properties. *Adv. Mater. Interfaces* **2020**, *7*, 2000484. DOI: 10.1002/admi.202000484
6. Chang, CC., Shih, JF., Chiou, YC., Lee, RT., Tseng, SF., Yang, CR. Development of textile-based triboelectric nanogenerators integrated with plastic metal electrodes for wearable devices. *Int. J. Adv. Manuf. Technol.* **2019**, *104*, 2633–2644. DOI: 10.1007/s00170-019-04160-9
7. Zhao, Y.; Wang, S.; Ding, Y.; Zhang, Z.; Huang, T.; Zhang, Y.; Wan, X.; Wang, Z. L.; Li, L. Piezotronic effect-augmented $Cu_{2-x}O$-$BaTiO_3$ sonosensitizers for multifunctional cancer dynamic therapy. *ACS Nano* **2022**, *16* (6), 9304–9316, DOI: 10.1021/acsnano.2c01968
8. Masekela, D.; Hintsho-Mbita, N. C.; Ntsendwana, B.; Mabuba, N. Thin films (FTO/$BaTiO_3$/AgNPs) for enhanced piezo-photocatalytic degradation of methylene blue and ciprofloxacin in wastewater. *ACS omega* **2022**, *7* (28), 24329–24343, DOI: 10.1021/acsomega.2c01699
9. Lin, P. Y.; Chen, K. H.; Miao, Y. B.; Chen, H. L.; Lin, K. J.; Chen, C. T.; Yeh, C. N.; Chang, Y.; Sung, H. W. Phase-changeable nanoemulsions for oral delivery of a therapeutic peptide: Toward targeting the pancreas for antidiabetic treatments using lymphatic transport. *Adv. Funct. Mater.* **2019**, *29* (13), 1809015, DOI: 10.1002/adfm.201809015

10. Zhang, F.; Challapalli, S. C.; Smith, P. J. Cannabinoid CB1 receptor activation stimulates neurite outgrowth and inhibits capsaicin-induced Ca^{2+} influx in an in vitro model of diabetic neuropathy. *Neuropharmacology* **2009**, *57* (2), 88–96, DOI: 10.1016/j.neuropharm.2009.04.017
11. Kim, T.; Kim, H. J.; Choi, W.; Lee, Y. M.; Pyo, J. H.; Lee, J.; Kim, J.; Kim, J.; Kim, J. H.; Kim, C. et al. Deep brain stimulation by blood-brain-barrier-crossing piezoelectric nanoparticles generating current and nitric oxide under focused ultrasound. *Nat. Biomed. Eng.* **2023**, *7* (2), 149–163, DOI: 10.1038/s41551-022-00965-4
12. Feifel, S. C.; Lisdat, F. Silica nanoparticles for the layer-by-layer assembly of fully electro-active cytochrome c multilayers. *J. Nanobiotechnol.* **2011**, *9* (1), 59, DOI: 10.1186/1477–3155-9-59
13. Nguyen, T. L.; Nam, Y. S.; Lee, S. Y.; Kim, H. C.; Jang, C. G. Effects of capsazepine, a transient receptor potential vanilloid type 1 antagonist, on morphine-induced antinociception, tolerance, and dependence in mice. *Br. J. Anaesth.* **2010**, *105* (5), 668–674, DOI: 10.1093/bja/aeq212
14. Soromou, L. W.; Jiang, L.; Wei, M.; Chen, N.; Huo, M.; Chu, X.; Zhong, W.; Wu, Q.; Baldé, A.; Deng, X. et al. Protection of mice against lipopolysaccharide-induced endotoxic shock by pinocembrin is correlated with regulation of cytokine secretion. *J. Immunotoxicol.* **2014**, *11* (1), 56–61, DOI: 10.3109/1547691X.2013.792886
15. Silva, J. F.; Olivon, V. C.; Mestriner, F. L. A. C.; Zanotto, C. Z.; Ferreira, R. G.; Ferreira, N. S.; Silva, C. A. A.; Luiz, J. P. M.; Alves, J. V.; Fazan, R. et al. Acute increase in O-GlcNAc improves survival in mice with LPS-induced systemic inflammatory response syndrome. *Front. Physiol.* **2020**, *10*, 1614, DOI: 10.3389/fphys.2019.01614
16. Wang, L.; Xu, J.; Xia, Y.; Yin, K.; Li, Z.; Li, B.; Wang, W.; Xu, H.; Yang, L.; Xu, Z. Muscarinic acetylcholine receptor 3 mediates vagus nerve-induced gastric cancer. *Oncogenesis* **2018**, *7* (11), 88, DOI: 10.1038/s41389-018-0099-6
17. Desrosiers-Gregoire, G.; Devenyi, G. A.; Grandjean, J.; Chakravarty, M. M. Rodent automated bold improvement of EPI sequences (RABIES): a standardized image processing and data quality platform for rodent fMRI. *bioRxiv* **2023**, 2022.08.20.504597, DOI: 10.1101/2022.08.20.504597
18. Grandjean J.; Desrosiers-Gregoire G.; Anckaerts C.; Angeles-Valdez D.; Ayad F.; Barrière D.A.; Blockx I.; Bortel A.; Broadwater M.; Cardoso B.M. A consensus protocol for functional connectivity analysis in the rat brain. *Nat. Neurosci.* **2023**, *26* (4), 673–681, DOI: 10.1038/s41593-023-01286-8
19. Qiu, L. R.; Fernandes, D. J.; Szulc-Lerch, K. U.; Dazai, J.; Nieman, B. J.; Turnbull, D. H.; Foster, J. A.; Palmert, M. R.; Lerch, J. P. Mouse MRI shows brain areas relatively larger in

males emerge before those larger in females. *Nat Commun*. **2018**, *9* (1), 2615, DOI: 10.1038/s41467-018-04921-2

20. Steadman, P. E.; Ellegood, J.; Szulc, K. U.; Turnbull, D. H.; Joyner, A. L.; Henkelman, R. M.; Lerch, J. P. Genetic effects on cerebellar structure across mouse models of autism using a magnetic resonance imaging atlas. *Autism Res*. **2014**, *7* (1), 124–37, DOI: 10.1002/aur.1344
21. Ullmann, J. F. P.; Watson, C.; Janke, A. L.; Kurniawan, N. D.; Reutens, D. C. A. segmentation protocol and MRI atlas of the C57BL/6J mouse neocortex. *Neuroimage*. **2013**, *78*, 196–203, DOI: 10.1016/j.neuroimage.2013.04.008
22. Richards, K.; Watson, C.; Buckley, R. F.; Kurniawan, N. D.; Yang, Z.; Keller, M. D.; Beare, R.; Bartlett, P. F.; Egan, G. F.; Galloway, G. J. et al. Segmentation of the mouse hippocampal formation in magnetic resonance images. *Neuroimage*. **2011**, *58* (3), 732–40, DOI: 10.1016/j.neuroimage.2011.06.025
23. Dorr, A. E.; Lerch, J. P.; Spring, S.; Kabani, N.; Henkelman, R. M; High resolution three-dimensional brain atlas using an average magnetic resonance image of 40 adult C57Bl/6J mice. *Neuroimage*. **2008**, *42* (1), 60–9, DOI: 10.1016/j.neuroimage.2008.03.037
24. Huston, J. M.; Ochani, M.; Rosas-Ballina, M.; Liao, H.; Ochani, K.; Pavlov, V. A.; Gallowitsch-Puerta, M.; Ashok, M.; Czura, C. J.; Foxwell, B. et al. Splenectomy inactivates the cholinergic antiinflammatory pathway during lethal endotoxemia and polymicrobial sepsis. *J. Exp. Med.* **2006**, *203*, 1623–1628, DOI: 10.1084/jem.20052362
25. Šestan, M.; Mikašinović, S.; Benić, A.; Wueest, S.; Dimitropoulos, C.; Mladenić, K.; Krapić, M.; Hiršl, L.; Glantzspiegel, Y.; Rasteiro, A. An IFNγ-dependent immune–endocrine circuit lowers blood glucose to potentiate the innate antiviral immune response. *Nat. Immunol.* **2024,** *25*, 981–993, DOI: 10.1038/s41590-024-01848-3
26. Cotero,V.; Fan, Y.; Tsaava, T.; Kressel, A. M.; Hancu, I.; Fitzgerald, P.; Wallace, K.; Kaanumalle, S.; Graf, J.; Rigby, W. et al. Noninvasive sub-organ ultrasound stimulation for targeted neuromodulation. *Nat. Commun.* **2019**, *10* (1), 952, DOI: 10.1038/s41467-019-08750-9
